# Supplementary figures and images for: Exploration of collective tactical variables in elite netball: An analysis of team and sub-group positioning behaviours
Source: PLoS One. 2024 Feb 26;19(2):e0295787. doi: 10.1371/journal.pone.0295787 (PMC10896551; doi:10.1371/journal.pone.0295787)

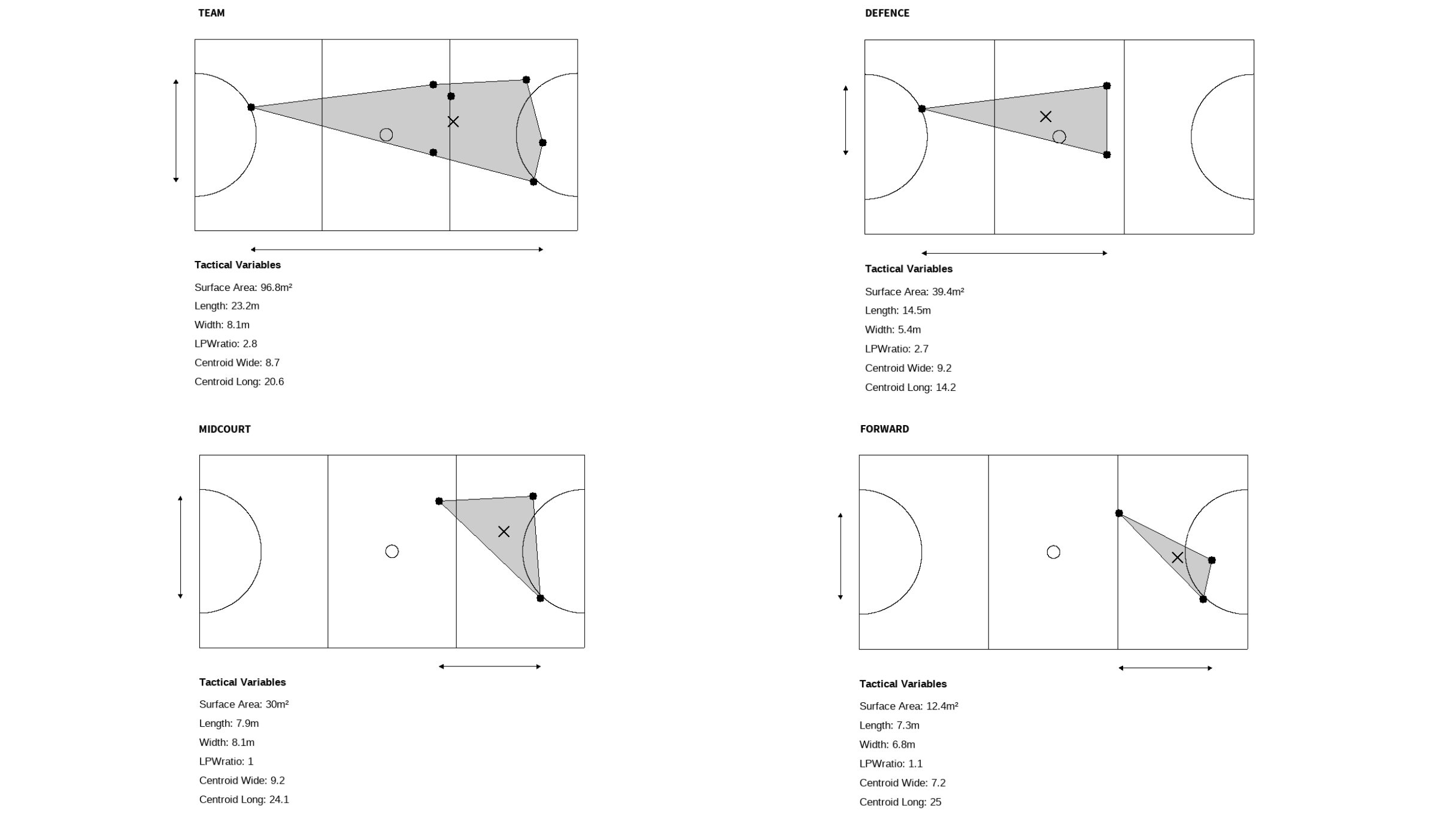

Supplement: S2 Video — For each sub-group. (GIF) [file pone.0295787.s002.gif]
