# Supplementary material for: Exploration of collective tactical variables in elite netball: An analysis of team and sub-group positioning behaviours
Source: PLoS One. 2024 Feb 26;19(2):e0295787. doi: 10.1371/journal.pone.0295787 (PMC10896551; doi:10.1371/journal.pone.0295787)
Supplement: S5 Table — Data shown are the range of the three correlations (mean, standard deviation and entropy). The variables have been ordered and outlined to show clusters with generally higher correlations between variables within the clusters than between the clusters. (PDF) [file pone.0295787.s007.pdf]

S5 Table. Correlations between collective tactical variables for the defender sub-group on attack. Data shown are the range of the three correlations (mean, standard deviation and entropy). The variables have been ordered and outlined to show clusters with generally higher correlations between variables within the clusters than between the clusters.

Variable	1	2	3	4	5	6	7	8	9	10
1. Stretch index		1.00, .98, .97	.92, .85, .87	.90, .81, .84	.79, .61, .71	.28, .22, .51	.28, .22, .50	-.27, -.13, -.36	.55, .36, .45	-.05, .12, .29
2. Inter-player distance	1.00, .98, .97		.92, .82, .84	.90, .82, .85	.80, .61, .71	.29, .25, .50	.30, .25, .49	-.27, -.13, -.36	.54, .37, .45	-.04, .12, .29
3. Stretch index longitudinal	.92, .85, .87	.92, .82, .84		.99, .93, .91	.64, .37, .56	-.05, .06, .42	-.04, .06, .40	-.53, -.18, -.41	.56, .39, .46	-.02, .15, .32
4. Length	.90, .81, .84	.90, .82, .85	.99, .93, .91		.60, .34, .52	-.08, .09, .38	-.08, .09, .36	-.57, -.21, -.39	.53, .34, .43	-.01, .14, .29
5. Surface area	.79, .61, .71	.80, .61, .71	.64, .37, .56	.60, .34, .52		.55, .47, .67	.55, .46, .66	.11, -.05, -.37	.53, .25, .46	-.04, .07, .37
6. Width	.28, .22, .51	.29, .25, .50	-.05, .06, .42	-.08, .09, .38	.55, .47, .67		.99, .96, .95	.75, -.29, -.55	.12, .31, .40	-.14, .34, .48
7. Stretch index lateral	.28, .22, .50	.30, .25, .49	-.04, .06, .40	-.08, .09, .36	.55, .46, .66	.99, .96, .95		.74, -.28, -.52	.12, .32, .40	-.16, .34, .45
8. Width per length ratio	-.27, -.13, -.36	-.27, -.13, -.36	-.53, -.18, -.41	-.57, -.21, -.39	.11, -.05, -.37	.75, -.29, -.55	.74, -.28, -.52		-.17, -.09, -.27	-.11, -.16, -.38
9. Centroid longitudinal	.55, .36, .45	.54, .37, .45	.56, .39, .46	.53, .34, .43	.53, .25, .46	.12, .31, .40	.12, .32, .40	-.17, -.09, -.27		-.02, .29, .36
10. Centroid lateral	-.05, .12, .29	-.04, .12, .29	-.02, .15, .32	-.01, .14, .29	-.04, .07, .37	-.14, .34, .48	-.16, .34, .45	-.11, -.16, -.38	-.02, .29, .36	

Uncertainty (90% compatibility limits): ~±0.1 to ~±0.01 for correlations of 0.00 to 0.95 respectively assuming a sample size of ~300.
