# Supplementary material for: Exploration of collective tactical variables in elite netball: An analysis of team and sub-group positioning behaviours
Source: PLoS One. 2024 Feb 26;19(2):e0295787. doi: 10.1371/journal.pone.0295787 (PMC10896551; doi:10.1371/journal.pone.0295787)
Supplement: S7 Table — With the exception of the mean centroid longitudinal and lateral, the statistics were derived via log-transformation, hence SDs are shown as times/divide factors. Clusters of variables representing consecutively longitudinal dispersion, lateral dispersion, longitudinal position and lateral position are outlined. (PDF) [file pone.0295787.s009.pdf]

S7 Table. Simple statistics provided by the mixed model (predicted mean for the middle of a match and mean possession duration; residual within-match standard deviation) for the team and positional sub-groups on attack. With the exception of the mean centroid longitudinal and lateral, the statistics were derived via log-transformation, hence SDs are shown as times/divide factors. Clusters of variables representing consecutively longitudinal dispersion, lateral dispersion, longitudinal position and lateral position are outlined.

Variable	Team	Forwards	Midcourts	Defenders
**Mean**				
Stretch index (m)	6.8 ×/÷ 1.07	3.3 ×/÷ 1.19	4.2 ×/÷ 1.16	4.7 ×/÷ 1.18
Inter-player distance (m)	10 ×/÷ 1.07	5.6 ×/÷ 1.19	7.4 ×/÷ 1.16	8.1 ×/÷ 1.19
Stretch index longitudinal (m)	6.1 ×/÷ 1.11	2.4 ×/÷ 1.30	3.2 ×/÷ 1.21	3.9 ×/÷ 1.28
Length (m)	22 ×/÷ 1.07	6.2 ×/÷ 1.30	8.1 ×/÷ 1.25	9.7 ×/÷ 1.26
Width (m)	7.3 ×/÷ 1.16	4.2 ×/÷ 1.26	5.6 ×/÷ 1.30	5.0 ×/÷ 1.27
Stretch index lateral (m)	2.0 ×/÷ 1.18	1.6 ×/÷ 1.29	2.2 ×/÷ 1.30	1.9 ×/÷ 1.27
Width per length ratio	0.30 ×/÷ 1.23	0.47 ×/÷ 1.76	0.53 ×/÷ 1.53	0.41 ×/÷ 1.54
Surface area (m^2)	86 ×/÷ 1.17	8.2 ×/÷ 1.51	15 ×/÷ 1.50	19 ×/÷ 1.51
Centroid longitudinal[a] (m)	18.3 ± 1.3	24.2 ± 2.1	19.0 ±1.3	11.5 ± 1.9
Centroid lateral (m)	7.5 ± 1.1	7.3 ± 1.4	7.7 ± 1.4	7.7 ± 1.2
**Variability**				
Stretch index (m)	0.80 ×/÷ 1.54	0.90 ×/÷ 1.42	0.99 ×/÷ 1.33	1.1 ×/÷ 1.48
Inter-player distance (m)	1.0 ×/÷ 1.44	1.6 ×/÷ 1.41	1.7 ×/÷ 1.40	1.8 ×/÷ 1.42
Stretch index longitudinal (m)	0.96 ×/÷ 1.52	0.93 ×/÷ 1.43	1.1 ×/÷ 1.43	1.1 ×/÷ 1.52
Length (m)	2.1 ×/÷ 1.43	2.4 ×/÷ 1.41	2.9 ×/÷ 1.31	2.5 ×/÷ 1.50
Width (m)	1.70 ×/÷ 1.33	1.8 ×/÷ 1.33	1.9 ×/÷ 1.45	1.6 ×/÷ 1.48
Stretch index lateral (m)	0.49 ×/÷ 1.52	0.68 ×/÷ 1.40	0.70 ×/÷ 1.40	0.62 ×/÷ 1.49
Width per length ratio	0.97 ×/÷ 1.69	0.58 ×/÷ 2.5	0.77 ×/÷ 2.12	0.79 ×/÷ 2.26
Surface area (m^2)	20 ×/÷ 1.41	6.6 ×/÷ 1.58	9.1 ×/÷ 1.57	10 ×/÷ 1.55
Centroid longitudinal (m)	2.0 ×/÷ 1.52	1.9 ×/÷ 1.42	2.5 ×/÷ 1.51	2.3 ×/÷ 1.49
Centroid lateral (m)	0.82 ×/÷ 1.55	1.2 ×/÷ 1.51	1.2 ×/÷ 1.59	0.87 ×/÷ 1.70
**Irregularity**				
Stretch index	0.19 ×/÷ 1.86	0.27 ×/÷ 1.56	0.25 ×/÷ 1.55	0.17 ×/÷ 1.70
Inter-player distance	0.20 ×/÷ 1.56	0.26 ×/÷ 1.51	0.25 ×/÷ 1.50	0.17 ×/÷ 1.69
Stretch index longitudinal	0.16 ×/÷ 1.87	0.25 ×/÷ 1.65	0.21 ×/÷ 1.54	0.15 ×/÷ 1.78
Length	0.20 ×/÷ 1.54	0.26 ×/÷ 1.50	0.22 ×/÷ 1.54	0.15 ×/÷ 1.83
Width	0.34 ×/÷ 1.44	0.34 ×/÷ 1.49	0.31 ×/÷ 1.39	0.28 ×/÷ 1.46
Stretch index lateral	0.31 ×/÷ 1.56	0.35 ×/÷ 1.46	0.31 ×/÷ 1.45	0.28 ×/÷ 1.45
Width per length ratio	0.25 ×/÷ 1.66	0.20 ×/÷ 1.74	0.17 ×/÷ 1.74	0.19 ×/÷ 1.72
Surface area	0.35 ×/÷ 1.48	0.34 ×/÷ 1.56	0.33 ×/÷ 1.55	0.24 ×/÷ 1.53
Centroid longitudinal	0.08 ×/÷ 1.86	0.12 ×/÷ 1.76	0.08 ×/÷ 1.83	0.10 ×/÷ 1.71
Centroid lateral	0.20 ×/÷ 1.47	0.21 ×/÷ 1.56	0.21 ×/÷ 1.50	0.20 ×/÷ 1.59

[a] Values shown were derived for log(30.5 – centroid longitudinal); actual means were therefore 18.6 m, 24.5 m, 19.1 m and 11.4 m respectively add actual means shown in output with decimal.
