# Supplementary material for: Exploration of collective tactical variables in elite netball: An analysis of team and sub-group positioning behaviours
Source: PLoS One. 2024 Feb 26;19(2):e0295787. doi: 10.1371/journal.pone.0295787 (PMC10896551; doi:10.1371/journal.pone.0295787)
Supplement: S8 Table — With the exception of the mean centroid longitudinal and lateral, the statistics were derived via log-transformation, hence SDs are shown as times/divide factors. Clusters of variables representing consecutively longitudinal dispersion, lateral dispersion, longitudinal position and lateral position are outlined. (PDF) [file pone.0295787.s010.pdf]

S8 Table. Simple statistics provided by the mixed model (predicted mean for the middle of a match and mean possession duration; residual within-match standard deviation) for the team and positional sub-groups on defence. With the exception of the mean centroid longitudinal and lateral, the statistics were derived via log-transformation, hence SDs are shown as times/divide factors. Clusters of variables representing consecutively longitudinal dispersion, lateral dispersion, longitudinal position and lateral position are outlined.

Variable	Team	Forwards	Midcourts	Defenders
**Mean**				
Stretch index (m)	6.6 ×/÷ 1.11	4.3 ×/÷ 1.17	3.9 ×/÷ 1.20	3.0 ×/÷ 1.20
Inter-player distance (m)	10 ×/÷ 1.09	7.5 ×/÷ 1.18	6.9 ×/÷ 1.20	5.1 ×/÷ 1.20
Stretch index longitudinal (m)	6.1 ×/÷ 1.14	3.8 ×/÷ 1.30	3.2 ×/÷ 1.26	2.2 ×/÷ 1.25
Length (m)	22 ×/÷ 1.08	9.5 ×/÷ 1.22	8.1 ×/÷ 1.24	5.9 ×/÷ 1.25
Width (m)	6.1 ×/÷ 1.18	3.8 ×/÷ 1.24	4.6 ×/÷ 1.28	3.7 ×/÷ 1.29
Stretch index lateral (m)	1.7 ×/÷ 1.19	1.5 ×/÷ 1.24	1.8 ×/÷ 1.28	1.4 ×/÷ 1.29
Width per length ratio	0.26 ×/÷ 1.22	0.34 ×/÷ 1.52	0.45 ×/÷ 1.49	0.49 ×/÷ 1.54
Surface area (m^2)	72 ×/÷ 1.17	14 ×/÷ 1.48	12 ×/÷ 1.54	7.0 ×/÷ 1.58
Centroid longitudinal[a] (m)	11.7 ± 1.7	18.9 ± 2.3	10.9 ± 1.6	5.8 ± 2.0
Centroid lateral (m)	7.5 ± 1.1	7.5 ± 1.2	7.4 ± 1.3	7.5 ± 1.3
**Variability**				
Stretch index (m)	0.89 ×/÷ 1.58	1.1 ×/÷ 1.42	1.0 ×/÷ 1.53	0.70 ×/÷ 1.50
Inter-player distance (m)	1.09 ×/÷ 1.45	1.8 ×/÷ 1.39	1.7 ×/÷ 1.51	1.2 ×/÷ 1.51
Stretch index longitudinal (m)	1.0 ×/÷ 1.55	1.1 ×/÷ 1.45	1.0 ×/÷ 1.52	0.73 ×/÷ 1.45
Length (m)	2.2 ×/÷ 1.48	2.6 ×/÷ 1.44	2.5 ×/÷ 1.52	1.9 ×/÷ 1.45
Width (m)	1.3 ×/÷ 1.43	1.3 ×/÷ 1.50	1.5 ×/÷ 1.41	1.4 ×/÷ 1.40
Stretch index lateral (m)	0.37 ×/÷ 1.44	0.50 ×/÷ 1.54	0.60 ×/÷ 1.37	0.55 ×/÷ 1.42
Width per length ratio	1.0 ×/÷ 1.70	0.68 ×/÷ 2.58	0.78 ×/÷ 2.28	0.65 ×/÷ 1.37
Surface area (m^2)	17 ×/÷ 1.48	7.9 ×/÷ 1.43	8.0 ×/÷ 1.64	4.7 ×/÷ 1.68
Centroid longitudinal (m)	1.50 ×/÷ 1.60	2.0 ×/÷ 1.42	2.4 ×/÷ 1.59	1.64 ×/÷ 1.43
Centroid lateral (m)	0.68 ×/÷ 1.61	0.76 ×/÷ 1.77	1.0 ×/÷ 1.60	0.94 ×/÷ 1.52
**Irregularity**				
Stretch index	0.16 ×/÷ 2.09	0.16 ×/÷ 1.65	0.22 ×/÷ 1.67	0.32 ×/÷ 1.57
Inter-player distance	0.17 ×/÷ 1.62	0.16 ×/÷ 1.80	0.23 ×/÷ 1.67	0.32 ×/÷ 1.62
Stretch index longitudinal	0.15 ×/÷ 2.02	0.15 ×/÷ 1.78	0.20 ×/÷ 1.64	0.28 ×/÷ 1.57
Length	0.18 ×/÷ 1.72	0.16 ×/÷ 1.83	0.22 ×/÷ 1.61	0.29 ×/÷ 1.54
Width	0.38 ×/÷ 1.44	0.32 ×/÷ 1.43	0.36 ×/÷ 1.35	0.39 ×/÷ 1.39
Stretch index lateral	0.37 ×/÷ 1.50	0.32 ×/÷ 1.40	0.36 ×/÷ 1.32	0.40 ×/÷ 1.37
Width per length ratio	0.33 ×/÷ 1.48	0.23 ×/÷ 1.70	0.21 ×/÷ 1.66	0.23 ×/÷ 1.73
Surface area	0.34 ×/÷ 1.46	0.27 ×/÷ 1.63	0.29 ×/÷ 1.52	0.39 ×/÷ 1.49
Centroid longitudinal	0.10 ×/÷ 1.98	0.12 ×/÷ 1.60	0.10 ×/÷ 1.87	0.12 ×/÷ 1.68
Centroid lateral	0.23 ×/÷ 1.61	0.23 ×/÷ 1.53	0.23 ×/÷ 1.53	0.26 ×/÷ 1.50

[a] Values shown were derived for log(30.5 – centroid longitudinal); actual means were therefore 18.6 m, 24.5 m, 19.1 m and 11.4 m respectively add actual means shown in output with decimal.
