# Supplementary material for: Exploration of collective tactical variables in elite netball: An analysis of team and sub-group positioning behaviours
Source: PLoS One. 2024 Feb 26;19(2):e0295787. doi: 10.1371/journal.pone.0295787 (PMC10896551; doi:10.1371/journal.pone.0295787)
Supplement: S9 Table — Dashed lines separate the variables into the clusters defined by the correlations in Table 2. With the exception of the mean centroid longitudinal and lateral, the statistics were derived via log-transformation, hence SDs are shown as percents. Data for attack and defence are predicted means (raw units) from the mixed model, and SDs are an appropriate residual representing differences between possessions. Data for attack minus defence are predicted mean differences (% units) with 90% compatibility limits (% units) and decisions about the magnitude of the differences. (PDF) [file pone.0295787.s011.pdf]

S9 Table. Collective tactical variables for the forward sub-group attack and defence possessions adjusted for possession duration, score difference, ladder point's difference, match trend and season trend. Dashed lines separate the variables into the clusters defined by the correlations in Table 2. With the exception of the mean centroid longitudinal and lateral, the statistics were derived via log-transformation, hence SDs are shown as percents. Data for attack and defence are predicted means (raw units) from the mixed model, and SDs are an appropriate residual representing differences between possessions. Data for attack minus defence are predicted mean differences (% units) with 90% compatibility limits (% units) and decisions about the magnitude of the differences.

**Variables**	**Attack**	**Defence**	**Attack minus Defence**	**Magnitude**
**Mean**				
Stretch index (m)	3.3 ± 19%	4.3 ± 17%	-28.8, ±8.9 %	**large**↓****
Inter-player distance (m)	5.6 ± 19%	7.5 ± 17%	-27.8, ±8.8 %	**large**↓****
Stretch index longitudinal (m)	2.39 ± 30%	3.75 ± 23%	-45, ±13 %	**large**↓****
Length (m)	6.2 ± 30%	9.5 ± 22%	-42, ±13 %	**large**↓****
Surface area (m^2)	8.2 ± 51%	13.5 ± 48%	-49, ±16 %	**moderate**↓****
Width (m)	4.2 ± 30%	3.83 ± 24%	9.8, ±7.4 %	small↑**
Stretch index lateral (m)	1.61 ± 29%	1.47 ± 24%	9.5, ±7.1 %	small↑**
Width per length ratio (m)	0.47 ± 76%	0.34 ± 52%	33, ±16 %	**moderate**↑***
Centroid longitudinal (m)	24.2 ± 2.1	18.9 ± 2.3	5.4, ±1.3	**very large**↑****
Centroid lateral (m)	7.29 ± 1.37	7.52 ± 1.21	-0.23, ±0.26	**small**↓*^{0}
**Variability**				
Stretch index (m)	0.90 ± 42%	1.06 ± 42%	-16.1, ±8.7 %	**small**↓***
Inter-player distance (m)	1.58 ± 41%	1.79 ± 40%	-12.7, ±7.5 %	**small**↓**
Stretch index longitudinal (m)	0.93 ± 43%	1.08 ± 54%	-15.1, ±8.2 %	**small**↓***
Length (m)	2.41 ± 41%	2.6 ± 44%	-7.5, ±5.9 %	**small**↓*^{0}
Surface area (m^2)	6.6 ± 58%	8.0 ± 43%	-19, ±10 %	**small**↓***
Width (m)	1.80 ± 33%	1.32 ± 50%	31, ±13 %	**moderate**↑****
Stretch index lateral (m)	0.68 ± 32%	0.50 ± 54%	30, ±13 %	**moderate**↑****
Width per length ratio (m)	0.58 ± 151%	0.67 ± 154%	-16, ±15 %	**trivial**↓^{0}*
Centroid longitudinal (m)	1.86 ± 50%	2.0 ± 42%	-6.7, ±9.7 %	trivial^{0}
Centroid lateral (m)	1.18 ± 51%	0.76 ± 77%	43.6, ±9.7 %	**moderate**↑****
**Irregularity**				
Stretch index	0.27 ± 56%	0.16 ± 76%	52, ±20 %	**moderate**↑****
Inter-player distance	0.26 ± 51%	0.16 ± 80%	65, ±28 %	**moderate**↑****
Stretch index longitudinal	0.25 ± 65%	0.15 ± 78%	54, ±21 %	**moderate**↑****
Length	0.26 ± 58%	0.16 ± 83%	52, ±19 %	**moderate**↑****
Surface area	0.34 ± 57%	0.27 ± 63%	22, ±12 %	**small**↑**
Width	0.34 ± 49%	0.32 ± 43%	8.2, ±7.9 %	**small**↑*^{0}
Stretch index lateral	0.35 ± 46%	0.32 ± 40%	8.5, ±7.9 %	**small**↑*^{0}
Width per length ratio	0.24 ± 74%	0.22 ± 70%	16, ±12 %	**small**↑*^{0}
Centroid longitudinal	0.12 ± 76%	0.12 ± 60%	4.7, ±14 %	trivial^{00}
Centroid lateral	0.21 ± 56%	0.23 ± 53%	-8, ±16 %	trivial↓^{0}*

90%CL, 90% compatibility limits; ↑, increase; ↓, decrease.

Magnitudes are based on the following scale for standardized changes in the mean: <0.2, trivial; 0.2-0.6, small; 0.6-1.2, moderate; 1.2-2.0, large; 2.0-4.0, very large; >4.0 extremely large

Reference-Bayesian likelihoods of substantial change: *possibly; **likely; ***very likely, ****most likely.

*** and **** indicate rejection of the non-superiority or non-inferiority hypothesis (p_{N-} or p_{N+} <0.05 and <0.005 respectively).

Reference-Bayesian likelihoods of trivial change: ^{0}possibly; ^{00}likely.

Likelihoods are not shown for effects with inadequate precision at the 90% level (failure to reject any hypotheses: p>0.05).

Effects in **bold** have adequate precision at the 99% level (p<0.005).
