# Supplementary material for: Exploration of collective tactical variables in elite netball: An analysis of team and sub-group positioning behaviours
Source: PLoS One. 2024 Feb 26;19(2):e0295787. doi: 10.1371/journal.pone.0295787 (PMC10896551; doi:10.1371/journal.pone.0295787)
Supplement: S12 Table — With the exception of the mean centroid longitudinal and lateral, the statistics were derived via log-transformation, hence data are the predicted changes (%, ±90% compatibility limits) and decisions about the magnitude of the changes. (PDF) [file pone.0295787.s014.pdf]

S12 Table. Difference between home score and home turnover (on attack) and between opposition score and opposition turnover (on defence) for each of the derived measures of each collective tactical variable for the forward sub-group. With the exception of the mean centroid longitudinal and lateral, the statistics were derived via log-transformation, hence data are the predicted changes (%, ±90% compatibility limits) and decisions about the magnitude of the changes.

Variables	Home (attack) Score – Turnover	Home (attack) Decision	Opposition (defence) Score – Turnover	Opposition (defence) Decision
**Mean**				
Stretch index (m)	-5.6, ±3.2 %	**small↓****	6.8, ±3.7 %	**small↑****
Inter-player distance (m)	-5.5, ±3.2 %	**small↓****	6.8, ±3.6 %	**small↑****
Stretch index longitudinal (m)	-7.8, ±4.6 %	**small↓****	9.8, ±4.9 %	**small↑****
Length (m)	-7.8, ±4.6 %	**small↓****	8.9, ±4.6 %	**small↑****
Surface area (m^2)	-8.8, ±8.2 %	**trivial↓**^{0*}	8.2, ±8.1 %	**trivial↑**^{0*}
Width (m)	-5.8, ±7.1 %	**trivial**^{00}	15, ±9.0 %	**small↑****
Stretch index lateral (m)	-0.80, ±4.3 %	trivial^{00}	-0.10, ±4.1 %	trivial^{000}
Width per length ratio (m)	9.6, ±9.9 %	**trivial↑**^{0*}	-7.6, ±6.9 %	**trivial↓**^{0*}
Centroid longitudinal (m)	0.77, ±0.36	**small↑****	-0.55, ±0.36	**small↓**^{*0}
Centroid lateral (m)	0.06, ±0.24	trivial^{00}	-0.15, ±0.20	**trivial**^{00}
**Variability**				
Stretch index (m)	-1.5, ±5.9 %	trivial^{00}	9.0, ±6.9 %	**small↑**^{*0}
Inter-player distance (m)	-2.6, ±5.7 %	**trivial**^{00}	9.3, ±6.9 %	**small↑**^{*0}
Stretch index longitudinal (m)	-0.50, ±6.1 %	trivial^{000}	11, ±7.2 %	**small↑****
Length (m)	-0.80, ±5.9 %	trivial^{000}	12, ±7.2 %	**small↑****
Surface area (m^2)	-2.7, ±14 %	trivial^{00}	-4.6, ±14 %	trivial^{00}
Width (m)	-4.9, ±7.6 %	**trivial**^{00}	7.3, ±7.6 %	**trivial↑**^{0*}
Stretch index lateral (m)	-6.7, ±5.3 %	**trivial↓**^{0*}	-4.3, ±6.5 %	**trivial**^{00}
Width per length ratio (m)	-26, ±11 %	**small↓****	-30, ±10 %	**small↓*****
Centroid longitudinal (m)	8.9, ±7.5 %	**small↑**^{*0}	6.6, ±6.9 %	**trivial↑**^{0*}
Centroid lateral (m)	-8.2, ±6.6 %	**trivial↓**^{0*}	-7.0, ±9.0 %	**trivial↓**^{0*}
**Irregularity**				
Stretch index	-4.7, ±7.0 %	**trivial**^{00}	-10, ±9.2 %	**small↓**^{*0}
Inter-player distance	-10, ±8.9 %	**trivial↓**^{0*}	8.3, ±10 %	**trivial↑**^{0*}
Stretch index longitudinal	-4.1, ±7.5 %	**trivial**^{00}	-9.1, ±9.5 %	**trivial↓**^{0*}
Length	-7.3, ±7.0 %	**trivial**^{00}	-12, ±9 %	**small↓**^{*0}
Surface area	5.0, ±8.0 %	**trivial**^{00}	-5.0, ±8.4 %	**trivial**^{00}
Width	-9.5, ±6.6 %	**small↓**^{*0}	-8.8, ±7.8 %	**trivial↓**^{0*}
Stretch index lateral	-2.8, ±6.1 %	**trivial**^{00}	-7.7, ±6.4 %	**trivial↓**^{0*}
Width per length ratio	12, ±11 %	**trivial**^{0*}	-7.7, ±8.8 %	**small↓**^{*0}
Centroid longitudinal	-5.6, ±3.2 %	**small↓****	6.8, ±3.7 %	**small↑****
Centroid lateral	-19, ±8.0 %	**small↓****	-3.5, ±9.3 %	**trivial**^{00}

↑, increase; ↓, decrease.

Magnitudes are based on the following scale for standardized changes in the mean: <0.2, trivial; 0.2-0.6, small; 0.6-1.2, moderate; 1.2-2.0, large; 2.0-4.0, very large; >4.0 extremely large

Reference-Bayesian likelihoods of substantial change: *possibly; **likely.

*** indicates rejection of the non-superiority or non-inferiority hypothesis (p_{N-} or p_{N+} <0.05).

Reference-Bayesian likelihoods of trivial change: ^{0}possibly; ^{00}likely; ^{000}very likely.

Likelihoods are not shown for effects with inadequate precision at the 90% level (failure to reject any hypotheses: p>0.05).

Effects in **bold** have adequate precision at the 99% level (p<0.005).
