# Supplementary material for: Exploration of collective tactical variables in elite netball: An analysis of team and sub-group positioning behaviours
Source: PLoS One. 2024 Feb 26;19(2):e0295787. doi: 10.1371/journal.pone.0295787 (PMC10896551; doi:10.1371/journal.pone.0295787)
Supplement: S16 Table — With the exception of the mean centroid longitudinal and lateral, the statistics were derived via log-transformation, hence data are the predicted changes (%, ±90% compatibility limits) and decisions about the magnitude of the changes. (PDF) [file pone.0295787.s018.pdf]

S16 Table. Effect of two SD of possession length (factor increases of 2.4 on attack and 2.7 on defence) on collective tactical variables for the forward's sub-group on attack and defence. With the exception of the mean centroid longitudinal and lateral, the statistics were derived via log-transformation, hence data are the predicted changes (%, ±90% compatibility limits) and decisions about the magnitude of the changes.

Variables	Attack	Decision	Defence	Decision
**Mean**				
Stretch index (m)	-2.6, ±3.2 %	**trivial^{0}**	13, ±4.3 %	**moderate↑******
Inter-player distance (m)	-3.0, ±3.1 %	**trivial↓^{0}***	12, ±4.2 %	**small↑******
Stretch index longitudinal (m)	-2.9, ±4.8 %	**trivial^{0}**	16, ±5.7 %	**small↑******
Length (m)	-3.6, ±4.7 %	**trivial↓^{0}***	13, ±5.3 %	**small↑*****
Surface area (m^2)	-4.7, ±7.2 %	**trivial^{0}**	23, ±11 %	**small↑*****
Width (m)	0.00, ±4.4 %	trivial^{0}	3.6, ±4.5 %	**trivial↑^{0}***
Stretch index lateral (m)	-0.10, ±4.2 %	trivial^{0}	3.4, ±4.6 %	**trivial↑^{0}***
Width per length ratio (m)	-3.8, ±8.4 %	**trivial^{00}**	-13, ±6.6 %	**small↓****
Centroid longitudinal (m)	0.58, ±0.33	**small↑*^{0}**	-0.66, ±0.37	**small↓****
Centroid lateral (m)	0.11, ±0.22	**trivial^{00}**	0.39, ±0.20	**small↑****
**Variability**				
Stretch index (m)	28, ±7.9 %	**moderate↑******	26, ±8.4 %	**moderate↑******
Inter-player distance (m)	26, ±7.8 %	**moderate↑******	23, ±8.3 %	**small↑******
Stretch index longitudinal (m)	27, ±7.9 %	**moderate↑******	27, ±8.3 %	**moderate↑******
Length (m)	25, ±7.7 %	**small↑******	23, ±8.2 %	**small↑******
Surface area (m^2)	14, ±8.7 %	**small↑****	34, ±11 %	**moderate↑******
Width (m)	18, ±6.5 %	**small↑******	26, ±8.7 %	**moderate↑******
Stretch index lateral (m)	17, ±6.2 %	**small↑*****	26, ±9.1 %	**moderate↑******
Width per length ratio (m)	-26, ±11 %	**small↓****	-30, ±10 %	**small↓*****
Centroid longitudinal (m)	27, ±9.1 %	**small↑******	27, ±8.9 %	**small↑******
Centroid lateral (m)	26, ±8.8 %	**small↑******	54, ±16 %	**moderate↑******
**Irregularity**				
Stretch index	-48, ±3.7 %	**large↓******	-42, ±6.4 %	**moderate↓******
Inter-player distance	-47, ±3.7 %	**moderate↓******	-45, ±6.5 %	**moderate↓******
Stretch index longitudinal	-52, ±3.5 %	**large↓******	-48, ±6.1 %	**moderate↓******
Length	-50, ±3.6 %	**large↓******	-46, ±5.8 %	**moderate↓******
Surface area	-43, ±3.8 %	**moderate↓******	-42, ±5.5 %	**moderate↓******
Width	-35, ±3.9 %	**moderate↓******	-41, ±4.6 %	**large↓******
Stretch index lateral	-35, ±3.8 %	**moderate↓******	-41, ±4.4 %	**large↓******
Width per length ratio	-75, ±18 %	**moderate↓******	-90, ±20 %	**moderate↓******
Centroid longitudinal	-49, ±5.1 %	**moderate↓******	-58, ±4.9 %	**large↓******
Centroid lateral	-40, ±4.4 %	**moderate↓******	-56, ±4.1 %	**large↓******

↑, increase; ↓, decrease.

Magnitudes are based on the following scale for standardized changes in the mean: <0.2, trivial; 0.2-0.6, small; 0.6-1.2, moderate; 1.2-2.0, large; 2.0-4.0, very large; >4.0 extremely large

Reference-Bayesian likelihoods of substantial change: *possibly; **likely; ***very likely, ****most likely.

*** and **** indicate rejection of the non-superiority or non-inferiority hypothesis (p_{N-} or p_{N+} <0.05 and <0.005 respectively).

Reference-Bayesian likelihoods of trivial change: ^{0}possibly.

Likelihoods are not shown for effects with inadequate precision at the 90% level (failure to reject any hypotheses: p>0.05).

Effects in **bold** have adequate precision at the 99% level (p<0.005).
