# Supplementary material for: Exploration of collective tactical variables in elite netball: An analysis of team and sub-group positioning behaviours
Source: PLoS One. 2024 Feb 26;19(2):e0295787. doi: 10.1371/journal.pone.0295787 (PMC10896551; doi:10.1371/journal.pone.0295787)
Supplement: S17 Table — With the exception of the mean centroid longitudinal and lateral, the statistics were derived via log-transformation, hence data are the predicted changes (%, ±90% compatibility limits) and decisions about the magnitude of the changes. (PDF) [file pone.0295787.s019.pdf]

S17 Table. Effect of two SD of possession length (factor increases of 2.4 on attack and 2.7 on defence) on collective tactical variables for the midcourt's sub-group on attack and defence. With the exception of the mean centroid longitudinal and lateral, the statistics were derived via log-transformation, hence data are the predicted changes (%, ±90% compatibility limits) and decisions about the magnitude of the changes.

Variables	Attack	Decision	Defence	Decision
**Mean**				
Stretch index (m)	7.2, ±2.5 %	**small↑*****	0.90, ±3.0 %	trivial^{00}
Inter-player distance (m)	7.0, ±2.5 %	**small↑*****	1.1, ±2.9 %	trivial^{00}
Stretch index longitudinal (m)	5.4, ±3.8 %	**small↑*^{0}**	-0.50, ±3.8 %	trivial^{00}
Length (m)	5.1, ±3.7 %	**small↑*^{0}**	-0.90, ±3.6 %	trivial^{00}
Surface area (m²)	22, ±7.3 %	**small↑******	14, ±8.7 %	**small↑****
Width (m)	8.1, ±4.0 %	**small↑****	4.7, ±5.0 %	**trivial^{0}***
Stretch index lateral (m)	10, ±4.2 %	**small↑*****	5.3, ±5.0 %	**trivial^{0}***
Width per length ratio (m)	0.60, ±6.7 %	trivial^{000}	7.5, ±7.6 %	**trivial^{0}***
Centroid longitudinal (m)	0.73, ±0.41	**small↑*^{0}**	-1.02, ±0.46	**small↓******
Centroid lateral (m)	0.10, ±0.20	**trivial^{00}**	0.51, ±0.24	**small↑****
**Variability**				
Stretch index (m)	26, ±8.2 %	**small↑******	24, ±9.9 %	**small↑******
Inter-player distance (m)	24, ±8.9 %	**small↑******	25, ±9.8 %	**small↑******
Stretch index longitudinal (m)	31, ±9.0 %	**moderate↑******	28, ±9.4 %	**small↑******
Length (m)	29, ±9.1 %	**small↑******	25, ±9.2 %	**small↑******
Surface area (m²)	28, ±9.9 %	**small↑******	18, ±11 %	**small↑****
Width (m)	28, ±8.0 %	**moderate↑******	18, ±8.1 %	**small↑*****
Stretch index lateral (m)	31, ±8.4 %	**moderate↑******	18, ±7.8 %	**small↑*****
Width per length ratio (m)	3.3, ±7.3 %	**trivial^{00}**	7.5 ±7.6 %	**trivial↑^{0}***
Centroid longitudinal (m)	37, ±8.9 %	**moderate↑******	37, ±11 %	**moderate↑******
Centroid lateral (m)	43, ±11 %	**moderate↑******	32, ±12 %	**small↑******
**Irregularity**				
Stretch index	-39, ±4.4 %	**moderate↓******	-50, ±4.7 %	**large↓******
Inter-player distance	-39, ±5.0 %	**moderate↓******	-52, ±4.3 %	**large↓******
Stretch index longitudinal	-47, ±4.1 %	**large↓******	-53, ±4.5 %	**large↓******
Length	-43, ±4.5 %	**moderate↓******	-53, ±4.8 %	**large↓******
Surface area	-38, ±3.3 %	**moderate↓******	-49, ±4.7 %	**large↓******
Width	-38, ±3.5 %	**large↓******	-38, ±3.9 %	**large↓******
Stretch index lateral	-40, ±3.4 %	**large↓******	-38, ±4.3 %	**large↓******
Width per length ratio	-43, ±5.8 %	**moderate↓******	-51, ±5.0 %	**moderate↓******
Centroid longitudinal	-48, ±5.2 %	**moderate↓******	-57, ±5.0 %	**large↓******
Centroid lateral	-40, ±4.9 %	**moderate↓******	-55, ±3.9 %	**large↓******

↑, increase; ↓, decrease.

Magnitudes are based on the following scale for standardized changes in the mean: <0.2, trivial; 0.2-0.6, small; 0.6-1.2, moderate; 1.2-2.0, large; 2.0-4.0, very large; >4.0 extremely large

Reference-Bayesian likelihoods of substantial change: *possibly; **likely; ***very likely, ****most likely.

*** and **** indicate rejection of the non-superiority or non-inferiority hypothesis (p_{N-} or p_{N+} <0.05 and <0.005 respectively).

Reference-Bayesian likelihoods of trivial change: ^{0}possibly; ^{00}likely; ^{000}very likely.

Likelihoods are not shown for effects with inadequate precision at the 90% level (failure to reject any hypotheses: p>0.05).

Effects in **bold** have adequate precision at the 99% level (p<0.005).
