# Supplementary material for: Exploration of collective tactical variables in elite netball: An analysis of team and sub-group positioning behaviours
Source: PLoS One. 2024 Feb 26;19(2):e0295787. doi: 10.1371/journal.pone.0295787 (PMC10896551; doi:10.1371/journal.pone.0295787)
Supplement: S18 Table — With the exception of the mean centroid longitudinal and lateral, the statistics were derived via log-transformation, hence data are the predicted changes (%, ±90% compatibility limits) and decisions about the magnitude of the changes. (PDF) [file pone.0295787.s020.pdf]

S18 Table. Effect of two SD of possession length (factor increases of 2.4 on attack and 2.7 on defence) on collective tactical variables for the defender's sub-group on attack and defence. With the exception of the mean centroid longitudinal and lateral, the statistics were derived via log-transformation, hence data are the predicted changes (%, ±90% compatibility limits) and decisions about the magnitude of the changes.

**Variables**	**Attack**	**Decision**	**Defence**	**Decision**
**Mean**				
Stretch index (m)	1.6, ±2.9 %	**trivial**^{00}	-5.2, ±3.3 %	**small**↓**
Inter-player distance (m)	1.3, ±2.8 %	**trivial**^{00}	-5.3, ±3.3 %	**small**↓**
Stretch index longitudinal (m)	2.1, ±3.9 %	**trivial**^{00}	-9.0, ±4.2 %	**small**↓**
Length (m)	-0.40, ±3.8 %	trivial^{000}	-9.5, ±4.2 %	**small**↓***
Surface area (m²)	9.3, ±7.6 %	**trivial**^{0*}	-4.0, ±8.5 %	trivial^{00}
Width (m)	4.6, ±4.4 %	**trivial**^{0*}	3.6, ±5.0 %	**trivial**^{00}
Stretch index lateral (m)	5.5, ±4.5 %	**trivial**^{0*}	3.6, ±4.9 %	**trivial**^{00}
Width per length ratio (m)	5.4, ±7.0 %	**trivial**^{00}	13, ±9.7 %	**small**↑*^{0}
Centroid longitudinal (m)	0.42, ±0.30	**trivial**^{00}	-0.80, ±0.33	**small**↓****
Centroid lateral (m)	0.10, ±0.20	**trivial**^{00}	0.33, ±0.21	**small**↑*^{0}
**Variability**				
Stretch index (m)	26, ±8.2 %	**small**↑****	28, ±9.9 %	**small**↑****
Inter-player distance (m)	23, ±8.8 %	**small**↑****	25, ±9.8 %	**small**↑****
Stretch index longitudinal (m)	31, ±9.0 %	**moderate**↑****	28, ±9.4 %	**small**↑****
Length (m)	28, ±9.0 %	**small**↑****	25, ±9.2 %	**small**↑****
Surface area (m²)	28, ±9.1 %	**small**↑****	18, ±11 %	**small**↑**
Width (m)	28, ±8.0 %	**moderate**↑****	16, ±7.6 %	**small**↑***
Stretch index lateral (m)	31, ±8.4 %	**moderate**↑****	14, ±6.9 %	**small**↑**
Width per length ratio (m)	-34, ±9.7 %	**small**↓****	-14, ±14 %	**trivial**↓^{0*}
Centroid longitudinal (m)	35, ±8.5 %	**moderate**↑****	36, ±11 %	**moderate**↑****
Centroid lateral (m)	43, ±11 %	**moderate**↑****	35, ±11 %	**moderate**↑****
**Irregularity**				
Stretch index	-45, ±5.2 %	**moderate**↓****	-52, ±3.7 %	**large**↓****
Inter-player distance	-48, ±4.8 %	**large**↓****	-53, ±3.6 %	**large**↓****
Stretch index longitudinal	-54, ±4.8 %	**large**↓****	-55, ±3.8 %	**large**↓****
Length	-43, ±4.1 %	**large**↓****	-52, ±3.8 %	**large**↓****
Surface area	-44, ±5.0 %	**moderate**↓****	-47, ±3.8 %	**large**↓****
Width	-44, ±3.4 %	**large**↓****	-40, ±4.1 %	**large**↓****
Stretch index lateral	-49, ±4.3 %	**large**↓****	-42, ±3.7 %	**large**↓****
Width per length ratio	-44, ±3.6 %	**large**↓****	-54, ±4.5 %	**large**↓****
Centroid longitudinal	-51, ±4.5 %	**large**↓****	-59, ±4.3 %	**large**↓****
Centroid lateral	-42, ±5.2 %	**moderate**↓****	-49, ±4.4 %	**large**↓****

↑, increase; ↓, decrease.

Magnitudes are based on the following scale for standardized changes in the mean: <0.2, trivial; 0.2-0.6, small; 0.6-1.2, moderate; 1.2-2.0, large; 2.0-4.0, very large; >4.0 extremely large

Reference-Bayesian likelihoods of substantial change: *possibly; **likely; ***very likely, ****most likely.

*** and **** indicate rejection of the non-superiority or non-inferiority hypothesis (p_{N-} or p_{N+} <0.05 and <0.005 respectively).

Reference-Bayesian likelihoods of trivial change: ^{0}possibly; ^{00}likely; ^{000}very likely.

Likelihoods are not shown for effects with inadequate precision at the 90% level (failure to reject any hypotheses: p>0.05).

Effects in **bold** have adequate precision at the 99% level (p<0.005).
