# Supplementary material for: Exploration of collective tactical variables in elite netball: An analysis of team and sub-group positioning behaviours
Source: PLoS One. 2024 Feb 26;19(2):e0295787. doi: 10.1371/journal.pone.0295787 (PMC10896551; doi:10.1371/journal.pone.0295787)
Supplement: S19 Table — With the exception of the mean centroid longitudinal and lateral, the statistics were derived via log-transformation, hence data are the predicted changes (%, ±90% compatibility limits) and decisions about the magnitude of the changes. (PDF) [file pone.0295787.s021.pdf]

S19 Table. Effect of a +10 points score difference on collective tactical variables for the team on attack and defence. With the exception of the mean centroid longitudinal and lateral, the statistics were derived via log-transformation, hence data are the predicted changes (%, ±90% compatibility limits) and decisions about the magnitude of the changes.

Variables	Attack	Decision	Defence	Decision
**Mean**				
Stretch index (m)	1.0, ±1.4 %	**trivial**^{00}	-0.60, ±2.2 %	trivial^{00}
Inter-player distance (m)	0.20, ±1.3 %	trivial^{00}	-1.0, ±1.9 %	**trivial↓**^{0*}
Stretch index longitudinal (m)	0.80, ±1.5 %	**trivial**^{00}	-0.7, ±2.7 %	trivial^{00}
Length (m)	-1.4, ±1.5 %	**trivial↓**^{0*}	-2.0, ±1.7 %	**small↓***^{0}
Width (m)	2.0, ±2.2 %	**trivial**^{00}	-0.1, ±3.0 %	trivial^{00}
Stretch index lateral (m)	1.8, ±2.9 %	trivial^{00}	-1.2, ±2.9 %	trivial^{00}
Width per length ratio (m)	4.7, ±3.6 %	**small↑***^{0}	0.80, ±4.3 %	**trivial**^{00}
Surface area (m^2)	0.60, ±2.9 %	trivial^{00}	-2.5, ±3.0 %	**trivial↓**^{0*}
Centroid longitudinal (m)	-0.13, ±0.31	**trivial**^{00}	0.18, ±0.35	trivial↑^{0*}
Centroid lateral (m)	0.19, ±0.23	**trivial↑**^{0*}	0.06, ±0.14	**trivial**^{000}
**Variability**				
Stretch index (m)	-5.9, ±5.8 %	**trivial↓**^{0*}	0.90, ±8.3 %	trivial^{00}
Inter-player distance (m)	-6.3, ±4.7 %	**trivial↓**^{0*}	1.7, ±8.6 %	trivial^{00}
Stretch index longitudinal (m)	-2.4, ±7.8 %	trivial^{00}	1.2, ±8.7 %	trivial^{00}
Length (m)	-8.4, ±6.5 %	**small↓***^{0}	1.1, ±8.0 %	trivial^{00}
Width (m)	-0.10, ±5.5 %	trivial^{000}	-1.1, ±6.2 %	trivial^{00}
Stretch index lateral(m)	0.10, ±5.8 %	trivial^{00}	-3.1, ±6.1 %	trivial^{00}
Width per length ratio (m)	11, ±7.9 %	**small↑***^{0}	-5.9, ±9.5 %	**trivial**^{00}
Surface area (m^2)	1.3, ±6.1 %	trivial^{00}	-6.1, ±4.8 %	**trivial↓**^{0*}
Centroid longitudinal (m)	5.2, ±8.6 %	**trivial**^{00}	17, ±9.2 %	**small↑****
Centroid lateral (m)	1.7, ±7.4 %	trivial^{000}	-2.0, ±9.3 %	trivial^{00}
**Irregularity**				
Stretch index	-1.6, ±8.3 %	trivial^{000}	5.3, ±8.6 %	**trivial**^{00}
Inter-player distance	-0.20, ±8.3 %	trivial^{000}	3.7, ±9.4 %	trivial^{00}
Stretch index longitudinal	0.60, ±9.9 %	trivial^{00}	6.8, ±8.5 %	**trivial**^{00}
Length	3.6, ±9.6 %	**trivial**^{00}	2.6, ±12 %	trivial^{00}
Width	-4.5, ±4.6 %	**trivial**^{00}	-1.8, ±4.9 %	**trivial**^{000}
Stretch index lateral	-3.0, ±4.6 %	**trivial**^{00}	0.40, ±4.4 %	**trivial**^{000}
Width per length ratio	1.6, ±6.8 %	**trivial**^{000}	13, ±9.2 %	**small↑****
Surface area	-11, ±5.5 %	**small↓****	0.80, ±6.2 %	trivial^{00}
Centroid longitudinal	-4.5, ±12 %	trivial^{00}	-16, ±13 %	**small↓***^{0}
Centroid lateral	-4.8, ±7.9 %	**trivial**^{00}	2.3, ±9.7 %	trivial^{00}

↑, increase; ↓, decrease.
Magnitudes are based on the following scale for standardized changes in the mean: <0.2, trivial; 0.2-0.6, small; 0.6-1.2, moderate; 1.2-2.0, large; 2.0-4.0, very large; >4.0 extremely large
Reference-Bayesian likelihoods of substantial change: *possibly; **likely.
Reference-Bayesian likelihoods of trivial change: ^{0}possibly; ^{00}likely; ^{000}very likely.
Likelihoods are not shown for effects with inadequate precision at the 90% level (failure to reject any hypotheses: p>0.05).
Effects in **bold** have adequate precision at the 99% level (p<0.005).
