# Supplementary material for: Exploration of collective tactical variables in elite netball: An analysis of team and sub-group positioning behaviours
Source: PLoS One. 2024 Feb 26;19(2):e0295787. doi: 10.1371/journal.pone.0295787 (PMC10896551; doi:10.1371/journal.pone.0295787)
Supplement: S20 Table — With the exception of the mean centroid longitudinal and lateral, the statistics were derived via log-transformation, hence data are the predicted changes (%, ±90% compatibility limits) and decisions about the magnitude of the changes. (PDF) [file pone.0295787.s022.pdf]

S20 Table. Effect of a +10 points score difference on collective tactical variables for the forward's sub-group on attack and defence. With the exception of the mean centroid longitudinal and lateral, the statistics were derived via log-transformation, hence data are the predicted changes (%, ±90% compatibility limits) and decisions about the magnitude of the changes.

Variables	Attack	Decision	Defence	Decision
**Mean**				
Stretch index (m)	-3.4, ±4.2 %	**trivial↓**^{0*}	-10, ±4.0 %	**small↓*****
Inter-player distance (m)	-3.7, ±4.2 %	**trivial↓**^{0*}	-9.6, ±3.9 %	**small↓*****
Stretch index longitudinal (m)	-7.1, ±6.0 %	**small↓**^{*0}	-12, ±5.1 %	**small↓*****
Length (m)	-8.1, ±5.8 %	**small↓****	-11, ±4.9 %	**small↓*****
Surface area (m²)	-3.6, ±9.3 %	trivial^{00}	-15, ±7.6 %	**small↓****
Width (m)	3.0, ±5.5 %	trivial↑^{0*}	0.00, ±3.3 %	trivial^{000}
Stretch index lateral (m)	3.1, ±5.3 %	trivial↑^{0*}	-0.50, ±3.4 %	trivial^{000}
Width per length ratio (m)	21, ±13 %	**small↑****	11, ±9.4 %	**small↑**^{*0}
Centroid longitudinal (m)	-0.33, ±0.48	**trivial↓**^{0*}	0.37, ±0.47	trivial↑^{0*}
Centroid lateral (m)	0.08, ±0.27	trivial^{00}	0.00, ±0.21	**trivial**^{000}
**Variability**				
Stretch index (m)	-11, ±6.1 %	**small↓****	-12, ±6.2 %	**small↓****
Inter-player distance (m)	-11, ±5.7 %	**small↓****	-11, ±5.7 %	**small↓****
Stretch index longitudinal (m)	-8.1, ±6.1 %	**small↓**^{*0}	-14, ±6.2 %	**small↓*****
Length (m)	-9.6, ±5.4 %	**small↓****	-14, ±4.6 %	**small↓*****
Surface area (m²)	-11, ±8.2 %	**small↓**^{*0}	-16, ±5.6 %	**small↓*****
Width (m)	-8.8, ±5.7 %	**small↓**^{*0}	-6.7, ±7.9 %	**trivial↓**^{0*}
Stretch index lateral (m)	-7.7, ±5.5 %	**small↓**^{*0}	-8.9, ±7.9 %	**small↓**^{*0}
Width per length ratio (m)	27, ±21 %	**small↑****	10, ±16 %	**trivial↑**^{0*}
Centroid longitudinal (m)	11, ±8.8 %	**small↑**^{*0}	14, ±7.7 %	**small↑****
Centroid lateral (m)	-3.0, ±6.5 %	**trivial**^{000}	4.5, ±11 %	trivial^{00}
**Irregularity**				
Stretch index	4.6, ±12.8 %	trivial^{00}	-2.7, ±9.2 %	trivial^{00}
Inter-player distance	6.1, ±12.6 %	trivial^{00}	-1.4, ±9.0 %	trivial^{00}
Stretch index longitudinal	9.3, ±13.8 %	**trivial↑**^{0*}	-8.3, ±9.1 %	**trivial↓**^{0*}
Length	7.5, ±13.1 %	**trivial↑**^{0*}	-7.1, ±8.8 %	**trivial↓**^{0*}
Surface area	-3.8, ±8.4 %	trivial^{00}	-9.1, ±8.2 %	**small↓**^{*0}
Width	4.5, ±5.9 %	**trivial**^{00}	-6.9, ±6.7 %	**trivial↓**^{0*}
Stretch index lateral	2.0, ±5.7 %	**trivial**^{000}	-8.2, ±6.5 %	**small↓**^{*0}
Width per length ratio	0.2, ±11 %	trivial^{00}	1.4, ±7.9 %	**trivial**^{000}
Centroid longitudinal	-8.6, ±8.8 %	**trivial↓**^{0*}	-22, ±9.1 %	**small↓*****
Centroid lateral	-7.3, ±10 %	**trivial↓**^{0*}	-5.6, ±8.2 %	**trivial**^{00}

↑, increase; ↓, decrease.

Magnitudes are based on the following scale for standardized changes in the mean: <0.2, trivial; 0.2-0.6, small; 0.6-1.2, moderate; 1.2-2.0, large; 2.0-4.0, very large; >4.0 extremely large

Reference-Bayesian likelihoods of substantial change: *possibly; **likely; ***very likely.

*** indicates rejection of the non-superiority or non-inferiority hypothesis (p_{N-} or p_{N+} <0.05).

Reference-Bayesian likelihoods of trivial change: ^{0}possibly; ^{00}likely; ^{000}very likely.

Likelihoods are not shown for effects with inadequate precision at the 90% level (failure to reject any hypotheses: p>0.05).

Effects in **bold** have adequate precision at the 99% level (p<0.005).
