# Supplementary material for: Exploration of collective tactical variables in elite netball: An analysis of team and sub-group positioning behaviours
Source: PLoS One. 2024 Feb 26;19(2):e0295787. doi: 10.1371/journal.pone.0295787 (PMC10896551; doi:10.1371/journal.pone.0295787)
Supplement: S21 Table — With the exception of the mean centroid longitudinal and lateral, the statistics were derived via log-transformation, hence data are the predicted changes (%, ±90% compatibility limits) and decisions about the magnitude of the changes. (PDF) [file pone.0295787.s023.pdf]

S21 Table. Effect of a +10 points score difference on collective tactical variables for the midcourt's sub-group on attack and defence. With the exception of the mean centroid longitudinal and lateral, the statistics were derived via log-transformation, hence data are the predicted changes (%, ±90% compatibility limits) and decisions about the magnitude of the changes.

Variables	Attack	Decision	Defence	Decision
**Mean**				
Stretch index (m)	6.7, ±2.7 %	**small↑*****	11, ±4.4 %	**moderate↑******
Inter-player distance (m)	6.9, ±2.7 %	**small↑*****	11, ±4.3 %	**moderate↑******
Stretch index longitudinal (m)	8.5, ±3.1 %	**small↑*****	13, ±5.7 %	**small↑*****
Length (m)	8.8, ±3.1 %	**small↑*****	13, ±5.5 %	**small↑******
Surface area (m^2)	9.8, ±6.9 %	**small↑*^{0}**	17, ±11 %	**small↑****
Width (m)	3.0, ±4.3 %	**trivial^{00}**	4.6, ±4.8 %	**trivial↑^{0}***
Stretch index lateral (m)	2.8, ±4.2 %	**trivial^{00}**	4.4, ±4.8 %	**trivial↑^{0}***
Width per length ratio (m)	-6.9, ±6.9 %	**trivial^{0}***	-2.7, ±7.0 %	trivial^{00}
Centroid longitudinal (m)	-0.89, ±0.58	**small↓****	0.96, ±0.59	**small↑*****
Centroid lateral (m)	0.05, ±0.23	trivial^{00}	0.19, ±0.19	**trivial^{00}**
**Variability**				
Stretch index (m)	-4.6, ±6.5 %	**trivial^{00}**	11, ±9.9 %	**small↑*^{0}**
Inter-player distance (m)	-4.9, ±6.7 %	**trivial^{00}**	11, ±9.7 %	**small↑*^{0}**
Stretch index longitudinal (m)	-4.3, ±7.8 %	trivial^{00}	14, ±9.9 %	**small↑****
Length (m)	-2.3, ±8.8 %	trivial^{00}	13.6, ±9.7 %	**small↑****
Surface area (m^2)	-2.2, ±9.3 %	trivial^{00}	6.5, ±12.6 %	trivial↑^{0}*
Width (m)	1.3, ±8.0 %	trivial^{00}	1.2, ±5.2 %	**trivial^{000}**
Stretch index lateral (m)	0.00, ±8.2 %	trivial	1.4, ±5.1 %	**trivial^{000}**
Width per length ratio (m)	-7.3, ±7.0 %	**trivial↓^{0}***	-2.9, ±7.0 %	trivial^{00}
Centroid longitudinal (m)	-4.9, ±7.7 %	**trivial^{00}**	5.9, ±8.8 %	**trivial↑^{0}***
Centroid lateral (m)	5.8, ±9.3 %	**trivial^{00}**	-6.5, ±6.1 %	**trivial^{00}**
**Irregularity**				
Stretch index	-6.1, ±8.4 %	**trivial↓^{0}***	-1.2, ±11 %	trivial^{00}
Inter-player distance	-7.6, ±8.1 %	**trivial↓^{0}***	-3.3, ±10 %	trivial^{00}
Stretch index longitudinal	-2.6, ±8.7 %	trivial^{00}	-0.1, ±11 %	trivial
Length	-3.0, ±8.7 %	trivial^{00}	-5.9, ±9.8 %	trivial↓^{0}*
Surface area	-2.8, ±7.0 %	trivial^{00}	5.8, ±8.5 %	**trivial^{00}**
Width	-8.1, ±5.7 %	**small↓*^{0}**	-11, ±6.7 %	**small↓****
Stretch index lateral	-6.2, ±5.6 %	**trivial↓^{0}***	-15, ±6.6 %	**small↓*****
Width per length ratio	9.6, ±12 %	**trivial↑^{0}***	-5.8, ±8.2 %	**trivial^{00}**
Centroid longitudinal	-1.3, ±10 %	trivial^{00}	-7.6, ±12 %	trivial↓^{0}*
Centroid lateral	-4.7, ±8.3 %	trivial^{00}	0.9, ±9.0 %	trivial^{00}

↑, increase; ↓, decrease.

Magnitudes are based on the following scale for standardized changes in the mean: <0.2, trivial; 0.2-0.6, small; 0.6-1.2, moderate; 1.2-2.0, large; 2.0-4.0, very large; >4.0 extremely large

Reference-Bayesian likelihoods of substantial change: *possibly; **likely; ***very likely, ****most likely.

*** and **** indicate rejection of the non-superiority or non-inferiority hypothesis (p_{N-} or p_{N+} <0.05 and <0.005 respectively).

Reference-Bayesian likelihoods of trivial change: ^{0}possibly; ^{00}likely; ^{000}very likely.

Likelihoods are not shown for effects with inadequate precision at the 90% level (failure to reject any hypotheses: p>0.05).

Effects in **bold** have adequate precision at the 99% level (p<0.005).
