# Supplementary material for: Exploration of collective tactical variables in elite netball: An analysis of team and sub-group positioning behaviours
Source: PLoS One. 2024 Feb 26;19(2):e0295787. doi: 10.1371/journal.pone.0295787 (PMC10896551; doi:10.1371/journal.pone.0295787)
Supplement: S22 Table — With the exception of the mean centroid longitudinal and lateral, the statistics were derived via log-transformation, hence data are the predicted changes (%, ±90% compatibility limits) and decisions about the magnitude of the changes. (PDF) [file pone.0295787.s024.pdf]

S22 Table. Effect of a +10 points score difference on collective tactical variables for the defender's sub-group on attack and defence. With the exception of the mean centroid longitudinal and lateral, the statistics were derived via log-transformation, hence data are the predicted changes (%, ±90% compatibility limits) and decisions about the magnitude of the changes.

Variables	Attack	Decision	Defence	Decision
**Mean**				
Stretch index (m)	-1.8, ±3.9 %	trivial⁰⁰	5.3, ±4.8 %	**small↑*⁰**
Inter-player distance (m)	-1.7, ±3.8 %	trivial⁰⁰	5.6, ±4.8 %	**small↑*⁰**
Stretch index longitudinal (m)	-2.1, ±5.2 %	trivial⁰⁰	7.0, ±6.5 %	**small↑*⁰**
Length (m)	-2.6, ±5.1 %	trivial⁰⁰	6.4, ±6.5 %	**small↑*⁰**
Surface area (m^2)	-5.1, ±9.2 %	trivial⁰⁰	8.5, ±12 %	**trivial⁰***
Width (m)	0.00, ±4.7 %	trivial⁰⁰	3.4, ±5.7 %	**trivial⁰***
Stretch index lateral (m)	-0.70, ±4.8 %	trivial⁰⁰	3.2, ±5.7 %	trivial⁰⁰
Width per length ratio (m)	3.3, ±8.8 %	trivial⁰⁰	-7.1, ±6.5 %	**trivial⁰***
Centroid longitudinal (m)	-0.66, ±0.43	**small↓*⁰**	0.39, ±0.43	**small↑*⁰**
Centroid lateral (m)	0.22, ±0.24	**trivial↑⁰***	0.10, ±0.18	**trivial⁰⁰**
**Variability**				
Stretch index (m)	-4.6, ±6.5 %	**trivial⁰⁰**	11, ±9.9 %	**small↑*⁰**
Inter-player distance (m)	-4.1, ±6.8 %	**trivial⁰⁰**	10, ±9.7 %	**small↑*⁰**
Stretch index longitudinal (m)	-4.3, ±7.8 %	trivial⁰⁰	14, ±9.9 %	**small↑****
Length (m)	-2.2, ±8.5 %	trivial⁰⁰	14, ±9.7 %	**small↑****
Surface area (m^2)	-0.60, ±9.1 %	trivial⁰⁰	6.5, ±13 %	trivial↑⁰*
Width (m)	1.3, ±8.0 %	trivial⁰⁰	1.2, ±5.1 %	**trivial⁰⁰⁰**
Stretch index lateral(m)	0.00, ±8.2 %	trivial	1.6, ±5.0 %	**trivial⁰⁰⁰**
Width per length ratio (m)	2.6, ±12 %	**trivial⁰⁰⁰**	1.2, ±13 %	trivial⁰⁰⁰
Centroid longitudinal (m)	-4.2, ±7.8 %	trivial⁰⁰	6.2, ±8.7 %	**trivial↑⁰***
Centroid lateral (m)	5.8, ±9.3 %	**trivial⁰⁰**	-5.0, ±6.0 %	**trivial⁰⁰**
**Irregularity**				
Stretch index	4.5, ±9.5 %	trivial⁰⁰	-13, ±8.5 %	**small↓****
Inter-player distance	2.2, ±9.2 %	trivial⁰⁰	-16, ±8.4 %	**small↓****
Stretch index longitudinal	11, ±8.6 %	**trivial↑⁰***	-10, ±9.4 %	**small↓*⁰**
Length	0.00, ±11 %	trivial	-16, ±8.5 %	**small↓****
Surface area	1.0, ±7.8 %	trivial⁰⁰	-6.2, ±6.9 %	**trivial↓⁰***
Width	-2.4, ±7.3 %	trivial⁰⁰	-6.9, ±4.4 %	**trivial↓⁰***
Stretch index lateral	-1.9, ±7.5 %	trivial⁰⁰	-7.1, ±4.3 %	**trivial↓⁰***
Width per length ratio	3.8, ±9.3 %	trivial⁰⁰	0.9, ±11 %	trivial⁰⁰
Centroid longitudinal	-0.3, ±9.1 %	trivial⁰⁰⁰	-5.6, ±12 %	trivial⁰⁰
Centroid lateral	-7.6, ±7.6 %	**trivial↓⁰***	-5.6, ±8.2 %	**trivial⁰⁰**

↑, increase; ↓, decrease.

Magnitudes are based on the following scale for standardized changes in the mean: <0.2, trivial; 0.2-0.6, small; 0.6-1.2, moderate; 1.2-2.0, large; 2.0-4.0, very large; >4.0 extremely large

Reference-Bayesian likelihoods of substantial change: *possibly; **likely.

Reference-Bayesian likelihoods of trivial change: ⁰possibly; ⁰⁰likely; ⁰⁰⁰very likely.

Likelihoods are not shown for effects with inadequate precision at the 90% level (failure to reject any hypotheses: p>0.05).

Effects in **bold** have adequate precision at the 99% level (p<0.005).
