# Supplementary material for: Exploration of collective tactical variables in elite netball: An analysis of team and sub-group positioning behaviours
Source: PLoS One. 2024 Feb 26;19(2):e0295787. doi: 10.1371/journal.pone.0295787 (PMC10896551; doi:10.1371/journal.pone.0295787)
Supplement: S23 Table — With the exception of the mean centroid longitudinal and lateral, the statistics were derived via log-transformation, hence data are the predicted changes (%, ±90% compatibility limits) and decisions about the magnitude of the changes. (PDF) [file pone.0295787.s025.pdf]

S23 Table. Change in collective tactical variables over a match for the team on attack and defence. With the exception of the mean centroid longitudinal and lateral, the statistics were derived via log-transformation, hence data are the predicted changes (%, ±90% compatibility limits) and decisions about the magnitude of the changes.

**Variables**	**Attack**	**Decision**	**Defence**	**Decision**
**Mean**				
Stretch index (m)	-2.2, ±2.2 %	**small**↓^{*0}	-7.9, ±2.9 %	**moderate**↓****
Inter-player distance (m)	-2.0, ±1.9 %	**small**↓^{*0}	-5.6, ±2.6 %	**moderate**↓***
Stretch index longitudinal (m)	-1.2, ±2.6 %	trivial↓^{0*}	-8.8, ±3.5 %	**moderate**↓****
Length (m)	-1.6, ±2.2 %	small↓^{*0}	-1.4, ±2.5 %	trivial↓^{0*}
Width (m)	-5.1, ±4.0 %	**small**↓**	-6.9, ±4.3 %	**small**↓**
Stretch index lateral (m)	-5.3, ±4.2 %	**small**↓**	-2.7, ±4.6 %	trivial↓^{0*}
Width per length ratio (m)	-6.4, ±5.5 %	**small**↓^{*0}	-3.6, ±5.9 %	trivial↓^{0*}
Surface area (m^2)	-5.4, ±4.3 %	**small**↓**	-7.4, ±4.4 %	**small**↓**
Centroid longitudinal (m)	0.51, ±0.45	**small**↑^{*0}	-0.80, ±0.50	**small**↓***
Centroid lateral (m)	-0.33, ±0.34	**small**↓^{*0}	-0.47, ±0.27	**small**↓**
**Variability**				
Stretch index (m)	10, ±12 %	**small**↑^{*0}	-7.3, ±12 %	trivial↓^{0*}
Inter-player distance (m)	7.8, ±11 %	**trivial**↑^{0*}	-11, ±12 %	small↓^{*0}
Stretch index longitudinal (m)	13, ±13 %	**small**↑^{*0}	-9.8, ±11 %	small↓^{*0}
Length (m)	7.3, ±12 %	trivial↑^{0*}	-7.8, ±11 %	small↓^{*0}
Width (m)	1.7, ±9.1 %	trivial	-20, ±8.1 %	**moderate**↓***
Stretch index lateral (m)	-1.2, ±9.1 %	trivial	-15, ±8.4 %	**small**↓**
Width per length ratio (m)	1.1, ±16 %	trivial	17, ±16 %	**small**↑**
Surface area (m^2)	-4.2, ±9.1 %	trivial↓^{0*}	-15, ±8.5 %	**small**↓**
Centroid longitudinal (m)	5.6, ±13 %	trivial↑^{0*}	-6.0, ±13 %	trivial↓^{0*}
Centroid lateral (m)	-12, ±11 %	**small**↓^{*0}	18, ±18 %	**small**↑**
**Irregularity**				
Stretch index	-8.1, ±13 %	trivial↓^{0*}	0.70, ±16 %	trivial
Inter-player distance	-8.2, ±12 %	trivial↓^{0*}	-1.7, ±15 %	trivial
Stretch index longitudinal	-8.8, ±13 %	trivial↓^{0*}	-9.6, ±14 %	trivial↓^{0*}
Length	6.2, ±15 %	trivial↑^{0*}	-10, ±15 %	small↓^{*0}
Width	-4.4, ±8.9 %	trivial↓^{0*}	8.1, ±11 %	small↑^{*0}
Stretch index lateral	-5.9, ±8.7 %	trivial↓^{0*}	2.3, ±8.9 %	trivial↑^{00}
Width per length ratio	0.00, ±13 %	trivial	-12, ±11 %	**small**↓^{*0}
Surface area	1.5, ±9.9 %	trivial	-12, ±9.4 %	**small**↓**
Centroid longitudinal	-9.5, ±18 %	trivial↓^{0*}	-3.4, ±21 %	trivial
Centroid lateral	26, ±16 %	**small**↑***	-13, ±12 %	**small**↓^{*0}

↑, increase; ↓, decrease.

Magnitudes are based on the following scale for standardized changes in the mean: <0.2, trivial; 0.2-0.6, small; 0.6-1.2, moderate; 1.2-2.0, large; 2.0-4.0, very large; >4.0 extremely large

Reference-Bayesian likelihoods of substantial change: *possibly; **likely; ***very likely, ****most likely.

*** and **** indicate rejection of the non-superiority or non-inferiority hypothesis (p_{N-} or p_{N+} <0.05 and <0.005 respectively).

Reference-Bayesian likelihoods of trivial change: ^{0}possibly; ^{00}likely.

Likelihoods are not shown for effects with inadequate precision at the 90% level (failure to reject any hypotheses: p>0.05).

Effects in **bold** have adequate precision at the 99% level (p<0.005).
