# Supplementary material for: Exploration of collective tactical variables in elite netball: An analysis of team and sub-group positioning behaviours
Source: PLoS One. 2024 Feb 26;19(2):e0295787. doi: 10.1371/journal.pone.0295787 (PMC10896551; doi:10.1371/journal.pone.0295787)
Supplement: S24 Table — With the exception of the mean centroid longitudinal and lateral, the statistics were derived via log-transformation, hence data are the predicted changes (%, ±90% compatibility limits) and decisions about the magnitude of the changes. (PDF) [file pone.0295787.s026.pdf]

S24 Table. Change in collective tactical variables over a match for the forward's sub-group on attack and defence. With the exception of the mean centroid longitudinal and lateral, the statistics were derived via log-transformation, hence data are the predicted changes (%, ±90% compatibility limits) and decisions about the magnitude of the changes.

Variables	Attack	Decision	Defence	Decision
**Mean**				
Stretch index (m)	-7.8, ±5.8 %	**small↓****	6.3, ±6.8 %	**small↑*⁰**
Inter-player distance (m)	-7.4, ±5.7 %	**small↓****	5.1, ±6.5 %	small↑*⁰
Stretch index longitudinal (m)	-5.5, ±8.7 %	small↓*⁰	6.8, ±8.8 %	small↑*⁰
Length (m)	-5.2, ±8.6 %	trivial↓⁰*	3.3, ±8.0 %	trivial↑⁰*
Surface area (m^2)	-9.0, ±13 %	small↓*⁰	22, ±17 %	**small↑****
Width (m)	-9.3, ±7.1 %	**small↓****	0.70, ±6.6 %	trivial
Stretch index lateral (m)	-9.6, ±6.8 %	**small↓****	1, ±6.7 %	trivial
Width per length ratio (m)	-8.1, ±15 %	trivial↑⁰*	0.4, ±13 %	trivial
Centroid longitudinal (m)	-0.7, ±0.68	trivial	-0.38, ±0.65	small↓*⁰
Centroid lateral (m)	-0.26, ±0.41	trivial↓⁰*	-0.42, ±0.33	**small↓****
**Variability**				
Stretch index (m)	1.4, ±10 %	trivial	28, ±14 %	**moderate↑*****
Inter-player distance (m)	2.8, ±10 %	trivial↑	25, ±13%	**small↑*****
Stretch index longitudinal (m)	2.5, ±11 %	trivial	24, ±14 %	**small↑*****
Length (m)	5.2, ±10 %	trivial↑⁰*	17, ±12 %	**small↑****
Surface area (m^2)	1.5, ±14 %	trivial	14, ±13 %	**small↑*⁰**
Width (m)	2.5, ±9.9 %	trivial⁰⁰	-11, ±11 %	**small↓*⁰**
Stretch index lateral (m)	2.0, ±9.5 %	trivial⁰⁰	-8.8, ±11 %	small↓*⁰
Width per length ratio (m)	-3.1 ±24 %	trivial	4.9, ±25 %	trivial
Centroid longitudinal (m)	4.9, ±13 %	trivial↑⁰*	-10, ±9.8 %	**small↓*⁰**
Centroid lateral (m)	-2.4, ±12 %	trivial⁰⁰	7.8, ±18 %	trivial↑⁰*
**Irregularity**				
Stretch index	-5.5, ±13 %	trivial↓⁰*	-33, ±12 %	**moderate↓******
Inter-player distance	-6.2, ±12 %	trivial↓⁰*	-30, ±13 %	**moderate↓*****
Stretch index longitudinal	1.4, ±15 %	trivial	-25, ±13 %	**small↓****
Length	2.9, ±14 %	trivial	-21, ±14 %	**small↓****
Surface area	-2.2, ±13 %	trivial	-21, ±11 %	**small↓****
Width	2.6, ±11 %	trivial	-1.7, ±11 %	trivial
Stretch index lateral	2.8, ±11 %	trivial	-2.2, ±11 %	trivial
Width per length ratio	-19, ±14 %	**small↓****	1.3, ±15 %	trivial
Centroid longitudinal	-11, ±16 %	trivial↓⁰*	5.6, ±17 %	trivial↑
Centroid lateral	16, ±15 %	**small↑*⁰**	-10, ±13 %	small↓*⁰

↑, increase; ↓, decrease.

Magnitudes are based on the following scale for standardized changes in the mean: <0.2, trivial; 0.2-0.6, small; 0.6-1.2, moderate; 1.2-2.0, large; 2.0-4.0, very large; >4.0 extremely large

Reference-Bayesian likelihoods of substantial change: *possibly; **likely; ***very likely, ****most likely.

*** and **** indicate rejection of the non-superiority or non-inferiority hypothesis (p_{N-} or p_{N+} <0.05 and <0.005 respectively).

Reference-Bayesian likelihoods of trivial change: ⁰possibly; ⁰⁰likely.

Likelihoods are not shown for effects with inadequate precision at the 90% level (failure to reject any hypotheses: p>0.05).

Effects in **bold** have adequate precision at the 99% level (p<0.005).
