# Supplementary material for: Exploration of collective tactical variables in elite netball: An analysis of team and sub-group positioning behaviours
Source: PLoS One. 2024 Feb 26;19(2):e0295787. doi: 10.1371/journal.pone.0295787 (PMC10896551; doi:10.1371/journal.pone.0295787)
Supplement: S25 Table — With the exception of the mean centroid longitudinal and lateral, the statistics were derived via log-transformation, hence data are the predicted changes (%, ±90% compatibility limits) and decisions about the magnitude of the changes. (PDF) [file pone.0295787.s027.pdf]

S25 Table. Change in collective tactical variables over a match for the midcourt's sub-group on attack and defence. With the exception of the mean centroid longitudinal and lateral, the statistics were derived via log-transformation, hence data are the predicted changes (%, ±90% compatibility limits) and decisions about the magnitude of the changes.

Variables	Attack	Decision	Defence	Decision
**Mean**				
Stretch index (m)	-12, ±3.6 %	**moderate↓******	-10, ±5.0 %	**moderate↓*****
Inter-player distance (m)	-12, ±3.6 %	**moderate↓******	-10, ±5.0 %	**moderate↓*****
Stretch index longitudinal (m)	-13, ±4.8 %	**moderate↓******	-13, ±6.3 %	**moderate↓*****
Length (m)	-13, ±4.9 %	**moderate↓******	-12, ±6.0 %	**small↓*****
Surface area (m^2)	-24, ±7.8 %	**moderate↓******	-15, ±11 %	**small↓****
Width (m)	-13, ±5.8 %	**small↓*****	-6.6, ±6.6 %	**small↓***⁰
Stretch index lateral (m)	-12, ±5.8 %	**small↓*****	-6.9, ±6.6 %	**small↓***⁰
Width per length ratio (m)	-0.90, ±12 %	trivial	5.6, ±12 %	trivial↑⁰*
Centroid longitudinal (m)	-0.29, ±0.82	trivial↓⁰*	0.20, ±0.81	trivial
Centroid lateral (m)	-0.02, ±0.38	trivial	-0.61, ±0.37	**small↓****
**Variability**				
Stretch index (m)	-21, ±8.4 %	**small↓*****	-19, ±11 %	**small↓****
Inter-player distance (m)	-22, ±8.8 %	**small↓*****	-19, ±11 %	**small↓****
Stretch index longitudinal (m)	-29, ±8.5 %	**moderate↓******	-16, ±11 %	**small↓****
Length (m)	-29, ±9.3 %	**moderate↓******	-11, ±11 %	**small↓***⁰
Surface area (m^2)	-9.0, ±13 %	trivial↓⁰*	-16, ±15 %	**small↓****
Width (m)	-5.2, ±11 %	trivial↓⁰*	-3.9, ±9.6 %	trivial↓⁰*
Stretch index lateral (m)	-2.6, ±12 %	trivial	-3.0, ±9.5 %	trivial⁰⁰
Width per length ratio (m)	-0.1, ±12 %	trivial	5.9, ±13 %	trivial↓⁰*
Centroid longitudinal (m)	9.2, ±13 %	small↑*⁰	-4.5, ±12 %	trivial↓⁰*
Centroid lateral (m)	-11, ±13 %	small↓*⁰	12, ±15 %	**small↑***⁰
**Irregularity**				
Stretch index	1.7, ±13 %	trivial	3.0, ±16 %	trivial
Inter-player distance	2.7, ±14 %	trivial	0.70, ±16 %	trivial
Stretch index longitudinal	11, ±15 %	small↑*⁰	1.4, ±16 %	trivial
Length	8.7, ±15 %	trivial↑⁰*	4.9, ±17 %	trivial
Surface area	-0.90, ±11 %	trivial	-5.8, ±13 %	trivial↓⁰*
Width	3.2, ±10 %	trivial	15, ±13 %	**small↑****
Stretch index lateral	4.3, ±10 %	trivial↑⁰*	22, ±14 %	**small↑*****
Width per length ratio	-10, ±16 %	trivial↓⁰*	1.0, ±17 %	trivial
Centroid longitudinal	-4.5, ±16 %	trivial	-4.8, ±18 %	trivial
Centroid lateral	12, ±15 %	small↑*⁰	2.6, ±14 %	trivial

↑, increase; ↓, decrease.

Magnitudes are based on the following scale for standardized changes in the mean: <0.2, trivial; 0.2-0.6, small; 0.6-1.2, moderate; 1.2-2.0, large; 2.0-4.0, very large; >4.0 extremely large

Reference-Bayesian likelihoods of substantial change: *possibly; **likely; ***very likely, ****most likely.

*** and **** indicate rejection of the non-superiority or non-inferiority hypothesis (p_{N-} or p_{N+} <0.05 and <0.005 respectively).

Reference-Bayesian likelihoods of trivial change: ⁰possibly; ⁰⁰likely.

Likelihoods are not shown for effects with inadequate precision at the 90% level (failure to reject any hypotheses: p>0.05).

Effects in **bold** have adequate precision at the 99% level (p<0.005).
