# Supplementary material for: Exploration of collective tactical variables in elite netball: An analysis of team and sub-group positioning behaviours
Source: PLoS One. 2024 Feb 26;19(2):e0295787. doi: 10.1371/journal.pone.0295787 (PMC10896551; doi:10.1371/journal.pone.0295787)
Supplement: S26 Table — With the exception of the mean centroid longitudinal and lateral, the statistics were derived via log-transformation, hence data are the predicted changes (%, ±90% compatibility limits) and decisions about the magnitude of the changes. (PDF) [file pone.0295787.s028.pdf]

S26 Table. Change in collective tactical variables over a match for the defender's sub-group on attack and defence. With the exception of the mean centroid longitudinal and lateral, the statistics were derived via log-transformation, hence data are the predicted changes (%, ±90% compatibility limits) and decisions about the magnitude of the changes.

Variables	Attack	Decision	Defence	Decision
**Mean**				
Stretch index (m)	-3.4, ±5.3 %	trivial↓^{0}*	-7.4, ±5.9 %	**small↓****
Inter-player distance (m)	-2.7, ±5.2 %	trivial↓^{0}*	-7.1, ±6.0 %	**small↓****
Stretch index longitudinal (m)	-3.7, ±7.2 %	trivial↓^{0}*	-9.6, ±7.8 %	**small↓****
Length (m)	-1.0, ±7.2 %	trivial	-8.9, ±7.9 %	**small↓****
Surface area (m²)	-0.10, ±14 %	trivial	-9.9, ±14 %	small↓*^{0}
Width (m)	1.4, ±7.5 %	trivial	-0.4, ±7.8 %	trivial
Stretch index lateral (m)	2.2, ±7.7 %	trivial	0.10, ±7.8 %	trivial
Width per length ratio (m)	1.1, ±14 %	trivial	17, ±16 %	**small↑****
Centroid longitudinal (m)	-0.06, ±0.61	trivial	0.65, ±0.60	small↑*^{0}
Centroid lateral (m)	-0.43, ±0.37	small↓**	-0.48, ±0.34	**small↓****
**Variability**				
Stretch index (m)	-21, ±8.4 %	**small↓*****	-19, ±11 %	**small↓****
Inter-player distance (m)	-22, ±8.5 %	**small↓*****	-19, ±11 %	**small↓****
Stretch index longitudinal (m)	-29, ±8.5 %	**moderate↓******	-16, ±11 %	**small↓****
Length (m)	-29, ±8.8 %	**moderate↓******	-11, ±11 %	**small↓*^{0}**
Surface area (m²)	-11, ±12 %	**small↓*^{0}**	-16, ±15 %	**small↓****
Width (m)	-5.2, ±11 %	trivial↓^{0}*	-5.3, ±9.3 %	trivial↓^{0}*
Stretch index lateral (m)	-2.6, ±12 %	trivial	-5.5, ±9.0 %	trivial↓^{0}*
Width per length ratio (m)	22, ±29 %	**small↑*^{0}**	13, ±29 %	trivial↑^{0}*
Centroid longitudinal (m)	7.8, ±13 %	trivial↑^{0}*	-4.1, ±12 %	trivial↓^{0}*
Centroid lateral (m)	-11, ±13 %	small↓*^{0}	10, ±14 %	**small↑*^{0}**
**Irregularity**				
Stretch index	18, ±18 %	**small↑****	23, ±18 %	**small↑****
Inter-player distance	18, ±18 %	**small↑****	23, ±18 %	**small↑****
Stretch index longitudinal	36, ±21 %	**small↑*****	10, ±17 %	trivial↑^{0}*
Length	55, ±26 %	**moderate↑******	10, ±16 %	trivial↑^{0}*
Surface area	-3, ±12 %	trivial	15, ±14 %	**small↑*^{0}**
Width	3.3, ±12 %	trivial	6.2, ±9.9 %	trivial↑^{0}*
Stretch index lateral	1.6, ±12 %	trivial	7.4, ±9.9 %	**trivial↑^{0}***
Width per length ratio	-20, ±13 %	**small↓****	3.6, ±18 %	trivial
Centroid longitudinal	-4.2, ±15 %	trivial^{00}	1.8, ±18 %	trivial
Centroid lateral	5.5, ±15 %	trivial↑^{0}*	2.6, ±14 %	trivial

↑, increase; ↓, decrease.

Magnitudes are based on the following scale for standardized changes in the mean: <0.2, trivial; 0.2-0.6, small; 0.6-1.2, moderate; 1.2-2.0, large; 2.0-4.0, very large; >4.0 extremely large

Reference-Bayesian likelihoods of substantial change: *possibly; **likely; ***very likely, ****most likely.

*** and **** indicate rejection of the non-superiority or non-inferiority hypothesis (p_{N-} or p_{N+} <0.05 and <0.005 respectively).

Reference-Bayesian likelihoods of trivial change: ^{0}possibly; ^{00}likely.

Likelihoods are not shown for effects with inadequate precision at the 90% level (failure to reject any hypotheses: p>0.05).

Effects in **bold** have adequate precision at the 99% level (p<0.005).
