# Supplementary material for: Exploration of collective tactical variables in elite netball: An analysis of team and sub-group positioning behaviours
Source: PLoS One. 2024 Feb 26;19(2):e0295787. doi: 10.1371/journal.pone.0295787 (PMC10896551; doi:10.1371/journal.pone.0295787)
Supplement: S27 Table — With the exception of the mean centroid longitudinal and lateral, the statistics were derived via log-transformation, hence data are the predicted changes (%, ±90% compatibility limits) and decisions about the magnitude of the changes. (PDF) [file pone.0295787.s029.pdf]

S27 Table. Effect of the strongest opposition minus the weakest opposition on collective tactical variables for the team on attack and defence. With the exception of the mean centroid longitudinal and lateral, the statistics were derived via log-transformation, hence data are the predicted changes (%, ±90% compatibility limits) and decisions about the magnitude of the changes.

Variables	Attack	Decision	Defence	Decision
**Mean**				
Stretch index (m)	-0.9, ±4.3 %	trivial	-5.9, ±11 %	moderate↓
Inter-player distance (m)	-0.4, ±4.1 %	trivial	-4.3, ±8.1 %	small↓
Stretch index longitudinal (m)	-0.9, ±3.5 %	trivial	-6.6, ±13 %	moderate↓
Length (m)	-2.0, ±5.9 %	small↓	-2.8, ±6.9 %	small↓
Width (m)	4.8, ±4.0 %	**small↑*⁰**	-3.7, ±8.3 %	small↓
Stretch index lateral (m)	2.2, ±8.3 %	trivial	-4.6, ±7.0 %	small↓*^{0}
Width per length ratio (m)	11, ±8.9 %	small↑**	0.10, ±17 %	trivial
Surface area (m^2)	3.0, ±8.9 %	trivial	-6.5, ±7.5 %	small↓**
Centroid longitudinal (m)	1.34, ±1.24	moderate↑**	-1.83, ±1.77	large↓**
Centroid lateral (m)	0.14, ±0.77	trivial	0.05, ±0.25	trivial^{00}
**Variability**				
Stretch index (m)	-17, ±12 %	small↓**	4.1, ±27 %	trivial
Inter-player distance (m)	-11, ±7.9 %	**small↓*^{0}**	5.1, ±27 %	trivial
Stretch index longitudinal (m)	-25, ±25 %	moderate↓**	-2.3, ±30 %	trivial
Length (m)	-4.4, ±20 %	trivial	3.1, ±23 %	trivial
Width (m)	-12, ±13 %	small↓**	-4.6, ±14 %	trivial
Stretch index lateral(m)	-11, ±15 %	small↓*^{0}	-6.1, ±15 %	trivial
Width per length ratio (m)	-1.9, ±25 %	trivial	7.2, ±11 %	trivial
Surface area (m^2)	-7.7, ±15 %	small↓	-8.5, ±8.4 %	**small↓*^{0}**
Centroid longitudinal (m)	-2.6, ±29 %	trivial	-24, ±14 %	moderate↓***
Centroid lateral (m)	-3.6, ±17 %	trivial	-3.9, ±27 %	trivial
**Irregularity**				
Stretch index	0.60, ±23 %	trivial	-27, ±28 %	moderate↓**
Inter-player distance	-2.2, ±22 %	trivial	-12, ±13 %	**small↓*^{0}**
Stretch index longitudinal	-2.3, ±34 %	trivial	16, ±63 %	small↑
Length	-6.4, ±27 %	trivial	-4.1, ±13 %	trivial
Width	3.2, ±8.6 %	trivial^{00}	-8.2, ±8.3 %	**small↓*^{0}**
Stretch index lateral	6.1, ±8.7 %	**trivial↑^{0}***	-6.3, ±29 %	trivial
Width per length ratio	-5.9, ±11 %	trivial↓^{0}*	-0.2, ±28 %	trivial
Surface area	4.2, ±17 %	trivial	-11, ±13 %	small↓*^{0}
Centroid longitudinal	-5.3, ±39 %	trivial	0.20, ±28 %	trivial
Centroid lateral	0.30, ±26 %	trivial	-16, ±17 %	small↓*^{0}

↑, increase; ↓, decrease.

Magnitudes are based on the following scale for standardized changes in the mean: <0.2, trivial; 0.2-0.6, small; 0.6-1.2, moderate; 1.2-2.0, large; 2.0-4.0, very large; >4.0 extremely large

Reference-Bayesian likelihoods of substantial change: *possibly; **likely; ***very likely, ****most likely.

*** indicates rejection of the non-superiority or non-inferiority hypothesis (p_{N-} or p_{N+} <0.05).

Reference-Bayesian likelihoods of trivial change: ^{0}possibly; ^{00}likely.

Likelihoods are not shown for effects with inadequate precision at the 90% level (failure to reject any hypotheses: p>0.05).

Effects in **bold** have adequate precision at the 99% level (p<0.005).
