# Supplementary material for: Exploration of collective tactical variables in elite netball: An analysis of team and sub-group positioning behaviours
Source: PLoS One. 2024 Feb 26;19(2):e0295787. doi: 10.1371/journal.pone.0295787 (PMC10896551; doi:10.1371/journal.pone.0295787)
Supplement: S28 Table — With the exception of the mean centroid longitudinal and lateral, the statistics were derived via log-transformation, hence data are the predicted changes (%, ±90% compatibility limits) and decisions about the magnitude of the changes. (PDF) [file pone.0295787.s030.pdf]

S28 Table. Effect of the strongest opposition minus the weakest opposition on collective tactical variables for the forward's sub-group on attack and defence. With the exception of the mean centroid longitudinal and lateral, the statistics were derived via log-transformation, hence data are the predicted changes (%, ±90% compatibility limits) and decisions about the magnitude of the changes.

Variables	Attack	Decision	Defence	Decision
**Mean**				
Stretch index (m)	5.1, ±21 %	small↑	1.2, ±23 %	trivial
Inter-player distance (m)	4.8, ±21 %	small↑	-0.10, ±22 %	trivial
Stretch index longitudinal (m)	10, ±32 %	small↑	1.3, ±33 %	trivial
Length (m)	9.0, ±31 %	small↑	-1.2, ±32 %	trivial
Surface area (m^2)	9.0, ±48 %	trivial	-5.0, ±29 %	trivial
Width (m)	0.50, ±24 %	trivial	-1.6, ±5.9 %	trivial^{00}
Stretch index lateral (m)	-0.30, ±23 %	trivial	-2.0, ±5.9 %	trivial^{00}
Width per length ratio (m)	1.0, ±56 %	trivial	1.3, ±28 %	trivial
Centroid longitudinal (m)	-0.11, ±3.41	trivial	-0.13, ±3.76	trivial
Centroid lateral (m)	0.19, ±0.78	trivial	-0.15, ±0.54	trivial
**Variability**				
Stretch index (m)	8.1, ±23 %	small↑	4.8, ±20 %	trivial
Inter-player distance (m)	8.1, ±21 %	small↑	3.0, ±16 %	trivial
Stretch index longitudinal (m)	-1.7, ±18 %	trivial	9.6, ±23 %	small↑
Length (m)	-2.8, ±14 %	trivial	6.5, ±10 %	trivial↑^{0*}
Surface area (m^2)	9.2, ±32 %	trivial	-4.0, ±15 %	trivial
Width (m)	-12, ±17 %	small↓^{*0}	3.0, ±39 %	trivial
Stretch index lateral (m)	-13, ±16 %	small↓**	1.4, ±40 %	trivial
Width per length ratio (m)	32, ±73 %	small↑	-1.8, ±33 %	trivial
Centroid longitudinal (m)	-19, ±21 %	small↓**	-16, ±16 %	small↓**
Centroid lateral (m)	-6.6, ±14 %	trivial↓^{0*}	11, ±30 %	trivial
**Irregularity**				
Stretch index	-18, ±33 %	small↓	-4.8, ±52 %	trivial
Inter-player distance	-15, ±36 %	small↓	-8.5, ±39 %	trivial
Stretch index longitudinal	7.1, ±43 %	trivial	-17, ±49 %	small↓
Length	-9.9, ±30 %	small↓	-18, ±42 %	small↓
Surface area	-17, ±36 %	small↓	8.3, ±27 %	trivial
Width	-0.60, ±22 %	trivial	-5.5, ±9.7 %	trivial↓^{0*}
Stretch index lateral	1.0, ±28 %	trivial	-2.1, ±9.8 %	trivial
Width per length ratio	-0.9,±28 %	trivial	12, ±16 %	**trivial↑^{0*}**
Centroid longitudinal	0.90, ±29 %	trivial	18, ±29 %	small↑^{*0}
Centroid lateral	-18, ±35 %	small↓	-12, ±41 %	small↓

↑, increase; ↓, decrease.
Magnitudes are based on the following scale for standardized changes in the mean: <0.2, trivial; 0.2-0.6, small; 0.6-1.2, moderate; 1.2-2.0, large; 2.0-4.0, very large; >4.0 extremely large
Reference-Bayesian likelihoods of substantial change: *possibly; **likely.
Reference-Bayesian likelihoods of trivial change: ^{0}possibly; ^{00}likely.
Likelihoods are not shown for effects with inadequate precision at the 90% level (failure to reject any hypotheses: p>0.05).
Effects in **bold** have adequate precision at the 99% level (p<0.005).
