# Supplementary material for: Exploration of collective tactical variables in elite netball: An analysis of team and sub-group positioning behaviours
Source: PLoS One. 2024 Feb 26;19(2):e0295787. doi: 10.1371/journal.pone.0295787 (PMC10896551; doi:10.1371/journal.pone.0295787)
Supplement: S29 Table — With the exception of the mean centroid longitudinal and lateral, the statistics were derived via log-transformation, hence data are the predicted changes (%, ±90% compatibility limits) and decisions about the magnitude of the changes. (PDF) [file pone.0295787.s031.pdf]

S29 Table. Effect of the strongest opposition minus the weakest opposition on collective tactical variables for the midcourt's sub-group on attack and defence. With the exception of the mean centroid longitudinal and lateral, the statistics were derived via log-transformation, hence data are the predicted changes (%, ±90% compatibility limits) and decisions about the magnitude of the changes.

Variables	Attack	Decision	Defence	Decision
**Mean**				
Stretch index (m)	-1, ±6.4 %	trivial	-8.6, ±18.4 %	small↓
Inter-player distance (m)	-0.3, ±6.5 %	trivial	-8.6, ±17.9 %	small↓
Stretch index longitudinal (m)	-2.3, ±4.9 %	trivial↓^{0}*	-11, ±22 %	small↓
Length (m)	-2.5, ±5 %	trivial↓^{0}*	-13, ±21 %	moderate↓
Surface area (m^2)	2.3, ±16 %	trivial	-3.4, ±39 %	trivial
Width (m)	8.5, ±13 %	small↑*^{0}	5.6, ±15 %	small↑
Stretch index lateral (m)	7.2, ±11 %	small↑*^{0}	5.8, ±15 %	small↑
Width per length ratio (m)	18, ±28 %	small↑**	27, ±28 %	small↑**
Centroid longitudinal (m)	0.16, ±3.92	trivial	0.06, ±4.75	trivial
Centroid lateral (m)	-0.14, ±0.61	trivial	0.28, ±0.34	**small↑*^{0}**
**Variability**				
Stretch index (m)	-13, ±16 %	small↓*^{0}	0.8, ±32 %	trivial
Inter-player distance (m)	-12, ±17 %	small↓*^{0}	-0.9, ±30 %	trivial
Stretch index longitudinal (m)	1.2, ±30 %	trivial	10, ±38 %	small↑
Length (m)	3.7, ±36 %	trivial	6.2, ±35 %	trivial
Surface area (m^2)	-17, ±33 %	small↓	2.9, ±51 %	trivial
Width (m)	-6.3, ±27 %	trivial	-6.8, ±8.6 %	**trivial↓^{0}***
Stretch index lateral (m)	-3.3, ±29 %	trivial	-6.7, ±8.4 %	**trivial↓^{0}***
Width per length ratio (m)	16, ±23 %	trivial	23, ±29 %	**small↑*^{0}**
Centroid longitudinal (m)	-6.2, ±28 %	trivial	3.2, ±35 %	trivial
Centroid lateral (m)	0.9, ±23 %	trivial	-6.6, ±11 %	trivial↓^{0}*
**Irregularity**				
Stretch index	11, ±36 %	small	5.8, ±37 %	trivial
Inter-player distance	16, ±35 %	small	7.3, ±44 %	trivial
Stretch index longitudinal	5.7, ±32 %	trivial	-15, ±35 %	small↓
Length	6.1, ±32 %	trivial	-17, ±15 %	small↓**
Surface area	5, ±22 %	trivial	5.1, ±43 %	trivial
Width	-2.9, ±17 %	trivial	-1.6, ±33 %	trivial
Stretch index lateral	4.5, ±15 %	trivial	-11, ±26 %	small↓
Width per length ratio	-20, ±25 %	small↓*^{0}	-3.6, ±15 %	trivial^{00}
Centroid longitudinal	-35, ±19 %	moderate↓***	3.7, ±16 %	trivial^{00}
Centroid lateral	3.7, ±27 %	trivial	2, ±38 %	trivial

↑, increase; ↓, decrease.

Magnitudes are based on the following scale for standardized changes in the mean: <0.2, trivial; 0.2-0.6, small; 0.6-1.2, moderate; 1.2-2.0, large; 2.0-4.0, very large; >4.0 extremely large

Reference-Bayesian likelihoods of substantial change: *possibly; **likely; ***very likely, ****most likely.

*** indicates rejection of the non-superiority or non-inferiority hypothesis (p_{N-} or p_{N+} <0.05).

Reference-Bayesian likelihoods of trivial change: ^{0}possibly; ^{00}likely.

Likelihoods are not shown for effects with inadequate precision at the 90% level (failure to reject any hypotheses: p>0.05).

Effects in **bold** have adequate precision at the 99% level (p<0.005).
