# Supplementary material for: Exploration of collective tactical variables in elite netball: An analysis of team and sub-group positioning behaviours
Source: PLoS One. 2024 Feb 26;19(2):e0295787. doi: 10.1371/journal.pone.0295787 (PMC10896551; doi:10.1371/journal.pone.0295787)
Supplement: S30 Table — With the exception of the mean centroid longitudinal and lateral, the statistics were derived via log-transformation, hence data are the predicted changes (%, ±90% compatibility limits) and decisions about the magnitude of the changes. (PDF) [file pone.0295787.s032.pdf]

S30 Table. Effect of the strongest opposition minus the weakest opposition on collective tactical variables for the defender's sub-group on attack and defence. With the exception of the mean centroid longitudinal and lateral, the statistics were derived via log-transformation, hence data are the predicted changes (%, ±90% compatibility limits) and decisions about the magnitude of the changes.

Variables	Attack	Decision	Defence	Decision
**Mean**				
Stretch index (m)	1.1, ±23 %	trivial	9.3, ±29 %	small↑
Inter-player distance (m)	1.0, ±24 %	trivial	8.1, ±29 %	small↑
Stretch index longitudinal (m)	-1.3, ±31 %	trivial	15, ±39 %	small↑
Length (m)	2.0, ±32 %	trivial	14, ±39 %	small↑
Surface area (m^2)	-9.7, ±51 %	small↓	2.4, ±58 %	trivial
Width (m)	1.2, ±13 %	trivial	-0.60, ±21 %	trivial
Stretch index lateral (m)	1.7, ±14 %	trivial	0.00, ±21 %	trivial
Width per length ratio (m)	-1.9, ±25 %	trivial	-14, ±11 %	**small↓****
Centroid longitudinal (m)	0.22, ±2.46	trivial	0.42, ±3.91	trivial
Centroid lateral (m)	0.11, ±0.77	trivial	0.25, ±0.31	trivial↑^{0}*
**Variability**				
Stretch index (m)	-13, ±16 %	small↓*^{0}	0.80, ±32 %	trivial
Inter-player distance (m)	-11, ±18 %	small↓	-0.90, ±30 %	trivial
Stretch index longitudinal (m)	1.2, ±30 %	trivial	10, ±38 %	small↑
Length (m)	2.9, ±38 %	trivial	6.2, ±35 %	trivial
Surface area (m^2)	-20, ±31 %	small↓	2.9, ±51 %	trivial
Width (m)	-6.3, ±27 %	trivial	-7.5, ±8.4 %	**small↓*^{0}**
Stretch index lateral (m)	-3.3, ±29 %	trivial	-8.4, ±8.0 %	**small↓*^{0}**
Width per length ratio (m)	1.3, ±22 %	trivial	-19, ±19 %	**small↓*^{0}**
Centroid longitudinal (m)	-7.4, ±29 %	trivial	4.0, ±35 %	trivial
Centroid lateral (m)	0.90, ±23 %	trivial	-3.8, ±19 %	trivial^{00}
**Irregularity**				
Stretch index	-16, ±19 %	small↓*^{0}	-17, ±32 %	small↓
Inter-player distance	-14, ±19 %	small↓*^{0}	-17, ±36 %	small↓
Stretch index longitudinal	-26, ±10 %	**small↓*****	-20, ±35 %	small↓
Length	-35, ±20 %	moderate↓***	-24, ±34 %	small↓
Surface area	7.9, ±22 %	trivial	-4.3, ±18 %	trivial
Width	-2.6, ±22 %	trivial	-1.8, ±8.5 %	trivial^{0}
Stretch index lateral	-2.1, ±22 %	trivial	-4.2, ±8.1 %	trivial↓^{0}*
Width per length ratio	22, ±24 %	small↑**	24, ±39 %	small↑*^{0}
Centroid longitudinal	-16, ±18 %	small↓*^{0}	-16, ±45 %	small↓
Centroid lateral	-8.1, ±19 %	trivial	-2.3, ±25 %	trivial

↑, increase; ↓, decrease.

Magnitudes are based on the following scale for standardized changes in the mean: <0.2, trivial; 0.2-0.6, small; 0.6-1.2, moderate; 1.2-2.0, large; 2.0-4.0, very large; >4.0 extremely large

Reference-Bayesian likelihoods of substantial change: *possibly; **likely; ***very likely, ****most likely.

*** indicates rejection of the non-superiority or non-inferiority hypothesis (p_{N-} or p_{N+} <0.05).

Reference-Bayesian likelihoods of trivial change: ^{0}possibly; ^{00}likely.

Likelihoods are not shown for effects with inadequate precision at the 90% level (failure to reject any hypotheses: p>0.05).

Effects in **bold** have adequate precision at the 99% level (p<0.005).
