# Supplementary material for: Exploration of collective tactical variables in elite netball: An analysis of team and sub-group positioning behaviours
Source: PLoS One. 2024 Feb 26;19(2):e0295787. doi: 10.1371/journal.pone.0295787 (PMC10896551; doi:10.1371/journal.pone.0295787)
Supplement: S31 Table — With the exception of the mean centroid longitudinal and lateral, the statistics were derived via log-transformation, hence data are the predicted changes (%, ±90% compatibility limits) and decisions about the magnitude of the changes. (PDF) [file pone.0295787.s033.pdf]

S31 Table. Change in collective tactical variables over the season for the team on attack and defence. With the exception of the mean centroid longitudinal and lateral, the statistics were derived via log-transformation, hence data are the predicted changes (%, ±90% compatibility limits) and decisions about the magnitude of the changes.

Variables	Attack	Decision	Defence	Decision
**Mean**				
Stretch index (m)	1.3, ±4.1 %	trivial	-3.5, ±10 %	small↓
Inter-player distance (m)	0.80, ±3.9 %	trivial	-2.3, ±7.6 %	small↓
Stretch index longitudinal (m)	0.20, ±3.4 %	trivial	-5.8, ±12 %	small↓
Length (m)	-2.4, ±5.5 %	small↓	-1.9, ±6.5 %	small↓
Width (m)	8.5, ±3.7 %	**small↑*******	8.1, ±8.7 %	small↑**
Stretch index lateral (m)	9.5, ±8.8 %	small↑**	9.2, ±7.4 %	small↑**
Width per length ratio (m)	14, ±8.6 %	moderate↑***	13, ±18 %	small↑**
Surface area (m²)	8.1, ±8.9 %	small↑**	5.3, ±7.8 %	small↑*^{0}
Centroid longitudinal (m)	0.15, ±1.14	trivial	-0.47, ±1.67	small↓
Centroid lateral (m)	-0.16, ±0.72	trivial	-0.08, ±0.23	trivial^{00}
**Variability**				
Stretch index (m)	-26, ±15 %	moderate↓***	-13, ±21 %	small↓
Inter-player distance (m)	-23, ±24 %	moderate↓**	-12, ±21 %	small↓
Stretch index longitudinal (m)	5.4, ±29 %	trivial	-11, ±26 %	small↓
Length (m)	-1.0, ±15 %	trivial	-8.4, ±19 %	small↓
Width (m)	-5.4, ±14 %	trivial	-11, ±13 %	small↓*^{0}
Stretch index lateral(m)	-2.6, ±16 %	trivial	-9.6, ±14 %	small↓*^{0}
Width per length ratio (m)	39, ±38 %	trivial	32, ±44 %	trivial↓^{0}*
Surface area (m²)	-23, ±11 %	moderate↓***	-11, ±7.4 %	**small↓****
Centroid longitudinal (m)	5.4, ±29 %	trivial	6.0, ±18 %	trivial
Centroid lateral (m)	-2.6, ±15 %	trivial	-4.2, ±25 %	trivial
**Irregularity**				
Stretch index	27, ±27 %	small↑**	31, ±18 %	**small↑*****
Inter-player distance	27, ±27 %	small↑**	25, ±24 %	small↑**
Stretch index longitudinal	28, ±41 %	small↑**	25, ±16 %	**small↑****
Length	36, ±37 %	small↑**	5.2, ±38 %	trivial
Width	7.7, ±8 %	**trivial↑^{0}***	7.6, ±8.8 %	**trivial↑^{0}***
Stretch index lateral	2.6, ±7.6 %	trivial^{00}	11, ±8.2 %	**small↑****
Width per length ratio	-13, ±9.1 %	**small↓****	-12, ±23 %	small↓
Surface area	6.4, ±17 %	trivial	12, ±15 %	small↑*^{0}
Centroid longitudinal	16, ±44 %	small↑	19, ±60 %	small↑
Centroid lateral	11, ±27 %	small↑	15, ±33 %	small↑

↑, increase; ↓, decrease.

Magnitudes are based on the following scale for standardized changes in the mean: <0.2, trivial; 0.2-0.6, small; 0.6-1.2, moderate; 1.2-2.0, large; 2.0-4.0, very large; >4.0 extremely large

Reference-Bayesian likelihoods of substantial change: *possibly; **likely; ***very likely, ****most likely.

*** and **** indicate rejection of the non-superiority or non-inferiority hypothesis (p_{N-} or p_{N+} <0.05 and <0.005 respectively).

Reference-Bayesian likelihoods of trivial change: ^{0}possibly; ^{00}likely.

Likelihoods are not shown for effects with inadequate precision at the 90% level (failure to reject any hypotheses: p>0.05).

Effects in **bold** have adequate precision at the 99% level (p<0.005).
