# Supplementary material for: Exploration of collective tactical variables in elite netball: An analysis of team and sub-group positioning behaviours
Source: PLoS One. 2024 Feb 26;19(2):e0295787. doi: 10.1371/journal.pone.0295787 (PMC10896551; doi:10.1371/journal.pone.0295787)
Supplement: S32 Table — With the exception of the mean centroid longitudinal and lateral, the statistics were derived via log-transformation, hence data are the predicted changes (%, ±90% compatibility limits) and decisions about the magnitude of the changes. (PDF) [file pone.0295787.s034.pdf]

S32 Table. Change in collective tactical variables over the season for the forward's sub-group on attack and defence. With the exception of the mean centroid longitudinal and lateral, the statistics were derived via log-transformation, hence data are the predicted changes (%, ±90% compatibility limits) and decisions about the magnitude of the changes.

Variables	Attack	Decision	Defence	Decision
**Mean**				
Stretch index (m)	5.8, ±20 %	small↑	-10, ±19 %	small↓
Inter-player distance (m)	5.8, ±20 %	small↑	-11, ±19 %	small↓
Stretch index longitudinal (m)	6.8, ±29 %	small↑	-15, ±25 %	small↓
Length (m)	5.1, ±28 %	trivial	-17, ±25 %	moderate↓
Surface area (m^2)	32, ±54 %	small↑	-3.6, ±27 %	trivial
Width (m)	7.6, ±24 %	small↑	9.5, ±6.0 %	**small**↑**
Stretch index lateral (m)	6.4, ±23 %	small↑	8.8, ±6.0 %	**small**↑**
Width per length ratio (m)	23, ±64 %	small↑	32, ±35 %	small↑**
Centroid longitudinal (m)	-0.85, ±3.15	small↓	0.62, ±3.53	small↑
Centroid lateral (m)	-0.22, ±0.73	trivial	-0.35, ±0.51	small↓*⁰
**Variability**				
Stretch index (m)	6.2, ±21 %	trivial	-6.6, ±17 %	trivial
Inter-player distance (m)	5.3, ±19 %	trivial	-8.3, ±13 %	small↓*⁰
Stretch index longitudinal (m)	-1.4, ±17 %	trivial	-8.2, ±18 %	small↓
Length (m)	-4.2, ±13 %	trivial	-11, ±7.8 %	**small**↓*⁰
Surface area (m^2)	21, ±33 %	small↑**	-5.7, ±13 %	trivial↓⁰*
Width (m)	-14, ±16 %	small↓**	-4.9, ±34 %	trivial
Stretch index lateral(m)	-15, ±14 %	small↓**	-7.2, ±34 %	trivial
Width per length ratio (m)	73, ±99 %	moderate↑**	33, ±42 %	small↑**
Centroid longitudinal (m)	-5.2, ±23 %	trivial	3.2, ±18 %	trivial
Centroid lateral (m)	-2.5, ±13 %	trivial	16, ±30 %	small↑
**Irregularity**				
Stretch index	-9.7, ±34 %	trivial	24, ±63 %	small↑
Inter-player distance	-8.3, ±37 %	trivial	26, ±49 %	small↑
Stretch index longitudinal	30, ±49 %	small↑**	17, ±64 %	small↑
Length	-13, ±27 %	small↓	18, ±56 %	small↑
Surface area	-9.7, ±36 %	trivial	15, ±27 %	small↑*⁰
Width	4.1, ±21 %	trivial	18, ±11 %	**small**↑**
Stretch index lateral	5.3, ±27 %	trivial	22, ±11 %	**small**↑***
Width per length ratio	-12, ±24 %	small	-4.6, ±12 %	trivial↓⁰⁰
Centroid longitudinal	14, ±30 %	small↑	12, ±25 %	trivial↑⁰*
Centroid lateral	-7.6, ±37 %	trivial	12, ±49 %	small↑

↑, increase; ↓, decrease.

Magnitudes are based on the following scale for standardized changes in the mean: <0.2, trivial; 0.2-0.6, small; 0.6-1.2, moderate; 1.2-2.0, large; 2.0-4.0, very large; >4.0 extremely large

Reference-Bayesian likelihoods of substantial change: *possibly; **likely; ***very likely.

*** indicates rejection of the non-superiority or non-inferiority hypothesis (p_{N-} or p_{N+} <0.05).

Reference-Bayesian likelihoods of trivial change: ⁰possibly; ⁰⁰likely.

Likelihoods are not shown for effects with inadequate precision at the 90% level (failure to reject any hypotheses: p>0.05).

Effects in **bold** have adequate precision at the 99% level (p<0.005).
