# Supplementary material for: Exploration of collective tactical variables in elite netball: An analysis of team and sub-group positioning behaviours
Source: PLoS One. 2024 Feb 26;19(2):e0295787. doi: 10.1371/journal.pone.0295787 (PMC10896551; doi:10.1371/journal.pone.0295787)
Supplement: S33 Table — With the exception of the mean centroid longitudinal and lateral, the statistics were derived via log-transformation, hence data are the predicted changes (%, ±90% compatibility limits) and decisions about the magnitude of the changes. (PDF) [file pone.0295787.s035.pdf]

S33 Table. Change in collective tactical variables over the season for the midcourt's sub-group on attack and defence. With the exception of the mean centroid longitudinal and lateral, the statistics were derived via log-transformation, hence data are the predicted changes (%, ±90% compatibility limits) and decisions about the magnitude of the changes.

Variables	Attack	Decision	Defence	Decision
**Mean**				
Stretch index (m)	6.4, ±6.6 %	small↑**	6.4, ±20 %	small↑
Inter-player distance (m)	7.6, ±6.7 %	small↑**	6.3, ±19 %	small↑
Stretch index longitudinal (m)	5.8, ±4.8 %	**small↑*^{0}**	3.0, ±24 %	trivial
Length (m)	7.6, ±4.9 %	**small↑****	3.3, ±23 %	trivial
Surface area (m^2)	6.5, ±16 %	trivial	11, ±41 %	small↑
Width (m)	9.4, ±12 %	small↑**	18, ±16 %	moderate↑**
Stretch index lateral (m)	8.3, ±11 %	small↑*^{0}	18, ±16 %	moderate↑**
Width per length ratio (m)	-1.0, ±23 %	trivial	20, ±25 %	small↑**
Centroid longitudinal (m)	-2.06, ±3.56	moderate↓	2.07, ±4.50	moderate↑
Centroid lateral (m)	-0.38, ±0.59	small↓*^{0}	0.12, ±0.31	trivial↑
**Variability**				
Stretch index (m)	-17, ±15 %	small↓**	21, ±36 %	small↑
Inter-player distance (m)	-16, ±15 %	small↓**	19, ±34 %	small↑
Stretch index longitudinal (m)	-19, ±23 %	small↓**	18, ±38 %	small↑
Length (m)	-15, ±27 %	small↓	17, ±36 %	small↑
Surface area (m^2)	-17, ±31 %	small↓	44, ±66 %	moderate↑**
Width (m)	-5.0, ±25 %	trivial	1.8, ±8.5 %	trivial^{00}
Stretch index lateral(m)	-4.4, ±27 %	trivial	3.2, ±8.5 %	trivial^{00}
Width per length ratio (m)	-3.1, ±21 %	trivial	16, ±19 %	small↑**
Centroid longitudinal (m)	-6.9, ±26 %	trivial	2.0, ±32 %	trivial
Centroid lateral (m)	-5.6, ±20 %	trivial	-7.5, ±10 %	**trivial↓^{0}***
**Irregularity**				
Stretch index	24, ±38 %	small↑**	28, ±48 %	small↑
Inter-player distance	21, ±34 %	small↑**	27, ±44 %	small↑
Stretch index longitudinal	25, ±35 %	small↑**	34, ±50 %	small↑**
Length	17, ±33 %	small↑	28, ±41 %	small↑**
Surface area	16, ±23 %	small↑*^{0}	30, ±21 %	small↑***
Width	2.0, ±16 %	trivial	-1.0, ±31 %	trivial
Stretch index lateral	2.6, ±14 %	trivial	-2.9, ±38 %	trivial
Width per length ratio	-7.6, ±27 %	trivial	-18, ±12 %	**small↓****
Centroid longitudinal	-4.8, ±26 %	trivial	2.9, ±40 %	trivial
Centroid lateral	5.1, ±26 %	trivial	18, ±32 %	small↑

↑, increase; ↓, decrease.

Magnitudes are based on the following scale for standardized changes in the mean: <0.2, trivial; 0.2-0.6, small; 0.6-1.2, moderate; 1.2-2.0, large; 2.0-4.0, very large; >4.0 extremely large

Reference-Bayesian likelihoods of substantial change: *possibly; **likely; ***very likely.

*** indicates rejection of the non-superiority or non-inferiority hypothesis (p_{N-} or p_{N+} <0.05 and <0.005 respectively).

Reference-Bayesian likelihoods of trivial change: ^{0}possibly; ^{00}likely.

Likelihoods are not shown for effects with inadequate precision at the 90% level (failure to reject any hypotheses: p>0.05).

Effects in **bold** have adequate precision at the 99% level (p<0.005).
