# Supplementary material for: Exploration of collective tactical variables in elite netball: An analysis of team and sub-group positioning behaviours
Source: PLoS One. 2024 Feb 26;19(2):e0295787. doi: 10.1371/journal.pone.0295787 (PMC10896551; doi:10.1371/journal.pone.0295787)
Supplement: S34 Table — With the exception of the mean centroid longitudinal and lateral, the statistics were derived via log-transformation, hence data are the predicted changes (%, ±90% compatibility limits) and decisions about the magnitude of the changes. (PDF) [file pone.0295787.s036.pdf]

S34 Table. Change in collective tactical variables over the season for the defender's sub-group on attack and defence. With the exception of the mean centroid longitudinal and lateral, the statistics were derived via log-transformation, hence data are the predicted changes (%, ±90% compatibility limits) and decisions about the magnitude of the changes.

Variables	Attack	Decision	Defence	Decision
**Mean**				
Stretch index (m)	-9.1, ±20 %	small↓	25, ±30 %	moderate↑**
Inter-player distance (m)	-8.6, ±20 %	small↓	24, ±31 %	moderate↑**
Stretch index longitudinal (m)	-14, ±25 %	small↓	30, ±41 %	moderate↑**
Length (m)	-14, ±25 %	moderate↓	30, ±41 %	moderate↑**
Surface area (m^2)	-15, ±44 %	small↓	57, ±83 %	moderate↑**
Width (m)	7.4, ±13 %	small↑*⁰	14, ±22 %	small↑
Stretch index lateral (m)	7.7, ±14 %	small↑*⁰	15, ±22 %	small↑**
Width per length ratio (m)	30, ±30 %	small↑**	-7.4, ±11 %	trivial⁰*
Centroid longitudinal (m)	-1.09, ±2.26	small↓	1.23, ±3.67	small↑
Centroid lateral (m)	-0.28, ±0.72	trivial	0.01, ±0.29	trivial
**Variability**				
Stretch index (m)	-17, ±15 %	small↓**	21, ±36 %	small↑
Inter-player distance (m)	-14, ±17 %	small↓**	19, ±34 %	small↑
Stretch index longitudinal (m)	-19, ±23 %	small↓**	18, ±38 %	small↑
Length (m)	-14, ±30 %	small↓	17, ±36 %	small↑
Surface area (m^2)	-16, ±30 %	small↓	44, ±66 %	moderate↑**
Width (m)	-5.0, ±25 %	trivial	2.3, ±8.5 %	trivial⁰⁰
Stretch index lateral (m)	-4.4, ±27 %	trivial	4.2, ±8.3 %	trivial
Width per length ratio (m)	29, ±25 %	**small↑*⁰**	17, ±25 %	**trivial↑⁰***
Centroid longitudinal (m)	-6.3, ±27 %	trivial	2.1, ±32 %	trivial
Centroid lateral (m)	-5.6, ±20 %	trivial	-7.1, ±9.6 %	**trivial↓⁰***
**Irregularity**				
Stretch index	7.4, ±23 %	trivial	-21, ±28 %	small↓
Inter-player distance	9.4, ±29 %	trivial	-28, ±29 %	moderate↓**
Stretch index longitudinal	13, ±14 %	**small↑*⁰**	-17, ±34 %	small↓
Length	14, ±33 %	small	-23, ±32 %	small↓
Surface area	3.5, ±19 %	trivial	-15, ±15 %	small↓**
Width	4.2, ±22 %	trivial	-6.8, ±7.3 %	**trivial↓⁰***
Stretch index lateral	1.5, ±21 %	trivial	-7.0, ±7.2 %	**trivial↓⁰***
Width per length ratio	-5.3, ±18 %	trivial	-7.7, ±27 %	trivial
Centroid longitudinal	-7.3, ±19 %	trivial	26, ±62 %	small
Centroid lateral	7.2, ±21 %	trivial	2.5, ±24 %	trivial

↑, increase; ↓, decrease.
Magnitudes are based on the following scale for standardized changes in the mean: <0.2, trivial; 0.2-0.6, small; 0.6-1.2, moderate; 1.2-2.0, large; 2.0-4.0, very large; >4.0 extremely large
Reference-Bayesian likelihoods of substantial change: *possibly; **likely.
Reference-Bayesian likelihoods of trivial change: ⁰possibly; ⁰⁰likely.
Likelihoods are not shown for effects with inadequate precision at the 90% level (failure to reject any hypotheses: p>0.05).
Effects in **bold** have adequate precision at the 99% level (p<0.005).
