# Supplementary material for: Exploration of collective tactical variables in elite netball: An analysis of team and sub-group positioning behaviours
Source: PLoS One. 2024 Feb 26;19(2):e0295787. doi: 10.1371/journal.pone.0295787 (PMC10896551; doi:10.1371/journal.pone.0295787)
Supplement: Attachment — Submitted filename: Response To Reviewers.docx [file pone.0295787.s037.docx]

**RESPONSE TO REVIEWERS**

| **REVIEWER 1** | | |
| --- | --- | --- |
| **Reviewer Statement** | **Response** | **Line #** |
| Line 30 to 31: “However, recent advancements in Local Positioning Systems (LPS) now provide an avenue to collect player tracking data indoors.”   - This sentence could be better supported by inserting some references | Have added in two references supporting the advancements in Local Positioning Systems | 30-31 |
| Line 32 to 34: “Tactical behaviour in sport can be defined as …”   - The authors are trying to define tactical behaviours from an ecological dynamics perspective, but I think this sentence omits important concepts like perception and action, and also the temporal nature of tactical behaviours. My understanding is that affordances arise from players’ perceptions of the environment, and tactical behaviours are the spontaneous actions performed in response to these dynamically changing affordances. Perhaps the authors may want to improve the definition along these lines. It might be good to also explain to the reader what affordances are. Also, if you are using the term ‘shared affordances’, I recommend referencing the study by Silva et al. (2013). | Added in the Silva (2013) reference to support. Have updated the text to explain players perceptions relationship with affordances and how opportunities to deal with tactical decision arise | 32-36 |
| Line 43 to 45: “The team centroid, …”  This description of team centroid could be sharpened. I suggest letting readers know that it is the mean x- and y-coordinate of a team’s players, and represents a central position of the team, when viewed as a unit (Sampaio & Maçãs, 2012). | Thanks good comment, have updated to explain the calculation and clear definition of the centroid | 46-48 |
| Line 45 to 47: “Research suggests that players are more coordinated with their position-specific centroid…”   - This assumes that readers automatically know what is position-specific centroid. I suggest first letting readers know that the concept of a centroid can also be used on a smaller group of players working more closely together, such as groups of defenders, midfielders, or forwards. Then you can go on to add findings from existing research, that players are more coordinated with these position-specific centroids… | Great comment, have provided an update explaining the centroid can also be used at a sub-group level | 50-53 |
| Line 57 to 63: “Team-level behaviours provide a global marker of performance, …”   - This paragraph is about examining tactical behaviours not only at team level, but also at sub-group levels. Since this is a main part of your paper (and also written in the title), I recommend devoting a few sentences telling the reader that this paradigm originated from theories in complex systems. You could also explain to the reader the concept of self-organisation, where order at a global level arises from component interactions at a lower level. Consider citing the work of Gréhaigne et al. (1997) who applied the concepts of systems and subsystems to team sports 25 years ago. For a contemporary example, consider also a recent study by Low et al. (2022), explaining to readers how sub-group analysis gives insights not observable at team level. | Have added the two references suggested. Also expanded on the concept of complex systems and how smaller sub-groups and can impact global complex systems. | 67-69 |
| Line 64 to 71: mean, variability, irregularity   - Although this paragraph is well-intentioned, it reads superficially. Other than saying that variability and irregularity are important, it does little to justify why so. Also, the last sentence tautologically says that irregularity gives insight to the regularity of a variable. Since nonlinearity is a characteristic of complex systems, consider explaining to the reader that behaviours with high irregularity (e.g. high ApEn) contain high complexity, and behaviours with high regularity (e.g. low ApEn) have low complexity, and are more predictable. Consider also explaining to readers the findings in regularity of two of the studies you cited (Aguiar et al., 2015; Gonçalves et al., 2016) | Added clear definition of Irregularity and regularity in regards to Approximate Entropy (ApEn) and used the references suggested. | 85-88 |
| Line 88 to 89: (31)   - Could you have meant to cite Aguiar et al. (2017) instead of Aguiar et al. (2015)? | Yes good pickup, have updated to the Aguiar et al. (2017) reference | 88 |
| Line 99: participants   - How many participants were there? Is it possible to give more information about them, e.g. height, weight, years of experience? | Added that there were 12 participants and included their height, weight and age information | 120 |
| Line 124: event data   - I appreciate the good effort that you put in, distinguishing analysis between attack and defence phases. Could you provide readers with intra-rater and inter-rater agreement scores, even if it is for a subset of the sample? | We have added intra – rater agreement scores which were previously conducted. However post-hoc inter-rater agreement testing is unviable as software required is expensive and not accessible currently. | 163 |
| Line 135 to 137: “Possession outcome was recorded as either a score or turnover…or other”   - Could you provide some descriptive statistics about how many possessions resulted in “score”, “turnover”, or “other” (for both analysed team and opponents)? | Added descriptive statistics regarding the number of possession for scores, turnovers and other for each turnover by team | 155 |
| Line 137 to 140: “Score difference…possession duration…ladder-points difference…”   - Could we see the distribution for these three data, either using a violin plot or histogram, perhaps in the supplementary section? | Included distribution graphs for possession length and score difference. As ladder-point’s difference is calculated before the match it is the same for each possession and thus only has 1 value per game and the distribution doesn’t provide any value due to the low number of samples. | Supplementary materials |
| Line 141: “…to account for…opposition strength differences…”   - Perhaps inform the reader what ladder position the analysed team finished at the end of the season? In the distribution of points between the best team and the worst team, where did the analysed team stand? | Added the top ranked, bottom ranked and analysed teams ladder points. | 165 |
| Line 147 to 148: “The origin for player position was set to one corner of the netball court. Attacking direction was reset each quarter as teams change the direction after each break.”   - Does the analysed team always attack from left to right? This has implications on centroid longitudinal. | Yes, good question. In our analysis and any accompanying visulisation. The attacking team attacks to the left. While the origin remains the same for analysis reasons, the 2nd and 4th quarters are inversed in their coordinates to ensure consistency in coordinate direction. | 170 |
| Line 154 to 155: “Additionally, when a player was substituted, the new player’s positional data were added to the previous player’s data at the point of substitution”   - Perhaps indicate the number of times that happened (n = XX)? | Added the count of times this occurred in the dataset | 178 |
| Line 160 to 161: “…forwards…midcourts…defenders…”   - I see that wing attack and wing defence are part of two sub-groups, which is fine by me. But what about centre, especially since they have the most degrees of freedom in movement areas, and arguably coordinate with the other two sub-groups as well? In any case consider talking about this in the discussion maybe? | Thanks for the great comment. With the main reasons being to keep each subgroup to the same number of player for consistency and from an netball expert perspectives, the centres while supporting both attack and defence doesn’t have priority in their attack attached to either of these subgroups like the WD and WA do. |  |
| Figures 2 to 5:   - Consider labelling the positions beside each dot. | Thanks for the suggestion, as these are collective variables from a specific point in time, we chose not to include player positions as these can swap and change within a second and don’t necessarily provide extra insight to the figures which are visualizing initial collective tactical variables |  |
| Line 191 to 192: “Mean distance of each player to all other players in the team or sub-group, including each player-player dyad only once in the calculation”   - Would be good to include in brackets the total number of pairs — I suppose 21? | Yes correct, 21 pairs. Have added this to the suggested text. | 215 |
| Line 194: “Average position of the team or sub-group of players”   - Since the word ‘position’ has been used to denote playing positions (i.e. wing attack, wing defence etc), may I suggest instead describing it as the average cartesian coordinates of the team / sub-group? | Great suggestion, have updated this to reflect the suggestion of the reviewer | 217 |
| Effect size   - Perhaps thresholds for magnitude effects should also be written in the main text (i.e. 0.2 small, 0.6 moderate, etc.) | Thanks for the suggestion, we thought it best to keep the heavy statistical analysis to the tables and statistical analysis section to ensure this paper was still digestible for non-statistical readers. |  |
| Table 3: “Dashed lines separate the variables…”   - Perhaps change the lines from solid to dashed? | Thanks have updated the text to be solid | Table 3 |
| Line 351: “…presenting opposite sign but similar magnitude…”   - I suggest explicitly telling the reader which sub-group had the positive sign and which sub-group had the negative sign. | Good suggestion, have added which sub-group the sign related to. | 375 |
| Line 367: “Positional sub-groups displayed varied effects of possession outcome.”   - Perhaps put in brackets the exact tables you are referencing (S12 to S14?) | Have referenced the supplementary tables S12 to S114 | 393 |
| Line 367 to 369: “The defender sub-group displayed the most dissimilar effects to that of the team, with its largest effects in the second cluster for mean, variability and irregularity.”   - In attack or in defence? | Added for both attack and defence. | 395 |
| Line 374: “The team (Figs 2-4)…”   - Do you mean (S15) instead? | Good pick up, have reflected this change | 401 |
| Line 377: “For the mean of the first cluster variables, the team and midcourt displayed small to moderate positive effects over the possession.”   - From what I see in S15 and S17, the mean for first cluster variables were trivial to small. | Yes good finding. Have reflected this change. | 405 |
| S15: centroid (longitudinal) in defence   - Sorry if I am wrong, but shouldn’t a value of -0.71 be moderate rather than small? | See table description, centroid longitudinal and lateral were not derived via log transformation, instead they are absolute values. |  |
| Figures 5 to 7   - Perhaps make them more reader-friendly by indicating left and right ends of the figures (higher in short possession, higher in long possession). | Thanks for the suggestion, have added reference to this in the Figure description in the manuscript. | Fig 5-7 |
| Line 396 to 397: “with the forward sub-group displaying the opposite sign to that of the midcourt and defender sub-groups”   - I think it is better to explicitly state which sub-group had the positive sign and which sub-group had the negative sign. | Made specific reference to which subgroups had what sign. | 432 |
| Line 400 to 402: “During defence, similar small to moderate effects were shown by the team and midcourt sub-group, but not the forward subgroup, as the defender sub-group displayed similar effects instead.”   - Better to be more precise (small to moderate decreases?). | Thanks, great suggestion. Have updated | 436 |
| Line 405 to 406: “Lastly, the midcourt sub-group displayed moderate effects for the second cluster of variables in defence.”   - From what I see in S25, they only displayed small decrease | Yes correct, updated | 442 |
| Line 408 to 410: “…only the variability in the second cluster variables had good evidence for a small reduction in lateral dispersion of the players when matched against a weaker team”   - Is it better to write surface area rather than lateral dispersion? | Have left as is, don’t want to confuse readers between the specific variable used in analyse surface area and surface area as a descriptive term |  |
| Line 411 to 412: “there was reasonable evidence for the defenders' small substantial reduction in longitudinal regularity when playing a weaker team”   - Please include the possession phase (during attack?). | Yes correct, during attack. Have updated. | 449 |
| Opening paragraph   - The discussion needs an opening paragraph which restates the research question and succinctly summarise the principal findings. In its current form, it begins rather abrupt. | Have added in an opening reflection of the main results and aim of the study | 466-469 |
| I suggest combining and shortening paragraphs (1), (2), and (3) for the following reasons: they take up one whole page while only citing one reference; the finding of correlated variables are not novel as Bartlett et al. (2012) already showed ten years ago. Thus, I think information here can be condensed, and it may be sufficient to tell the reader that   - your findings support previous research, and show that they can be extended to netball, and - researchers/practitioners only need selected variables (inter-player distance and width) that can adequately convey players’ movements longitudinally and laterally. | Thanks for the suggestion, we agree Bartlett has showed correlated variables, however we believe it is important to highlight the correlations for collective tactical variables in netball which have not been researched before. In regards to the second point, we also agree inter-player distance and width are the two more important lateral dispersion variables (holding the most practical information). We also believe it is worth covering the other variables in detail as it supports the use of width and inter-player distance as key variables for future research, and provides insight that other variables are maybe less practical moving forward (this is still an important finding) |  |
| At various points in the discussion, the authors make reference to movement restrictions of players in netball, particularly the GK and GS (lines 439, 443, 446, 478, 506).   - Could we draw parallels from related research regarding space restrictions e.g. Castellano et al. (2016); Gonçalves et al. (2017) or any other research that you know. Could we also draw parallels from the sport of football where studies calculating the team centroid frequently omit the goalkeepers, even though goalkeepers have no pitch-restrictions? | Thanks, this is a really good point. Have added the point regarding soccer/football goal keeper omission in calculation as this relates to the GK and GS in netball | 548-550 |
| Paragraph (9) variability and irregularity   - This paragraph is about variability and irregularity of the variables, and their relationships with possession duration. Can we make comparisons with the studies of Aguiar et al. (2017); Ric et al. (2016) or any other related study you know? | Thanks, these are both good references. Have updated to use both. | 556 |
| Paragraph (10) opposition strength   - Consider also making comparisons with Gonçalves et al. (2019) | Have added this in. | 558-559 |
| Paragraphs (12) and (13) — score status and match ID — make no comparisons with published research, and seem to merely explain the study’s results.   - What has research shown (in netball or other sports) about score status and tactical behaviours that are relevant to your study? | Thanks for this suggestion. Have added in a reference and further comment regarding the comparisons between other sports research and netball. | 580-583 |
| Line 440 to 441: “…length per width ratio…”   - Could you have meant width per length ratio? | Yes correct, have updated | 482 |
| Line 464 to 465: “The midcourt sub-group most epitomizes the use of lateral dispersion on attack…”   - Since midcourts seem more sensitive to width, can we draw comparisons with the study of Zubillaga et al. (2013) | Thanks for the suggestion, in this instances netballs unique positional restrictions is of focus and doesn’t compare with the openness soccer players have. |  |
| Line 487 to 488: “Thus, successful possessions may tend to be those with a more direct path to the goal”   - Is it then possible to retrospectively explore if successful possessions are shorter in duration (i.e. indeed more direct)? You could add this in the supplementary section if space is a concern. | Thanks for the suggestion, this global research suggestion doesn’t fit with the team and sub-group analysis. |  |
| Line 489 to 491: “When the opposition scored, the defender sub-group presented small reductions in inter-player distances, width and surface area, representing compactness of positioning, previously highlighted as a critical defensive strategy in netball”   - If compactness is critical, why then did opponents still score? Could you also compare it with studies like Moura et al. (2012)? | Have updated to clarify, compactness is a sought after defensive strategy, however this can be more effective against slower teams and not on turnover possessions | 535-536 |
| Line 502 to 503: “…Santos et al., 2018…”   - This citation seems to be in a different format | Updated |  |
| Line 522 to 523: “…as previous literature in soccer has suggested that tactics and group behaviours over the course of a season change”   - Could you give citations for these literature? | Thanks, there is a citation at the end of the sentence, (11). | 572 |
| Line 537: limitation / future directions   - I believe data only came from one analysed team, and only home matches were analysed. Perhaps these could also be listed as limitations. | Thanks, good point. Have updated to reflect this. | 598 |
