# Supplementary material for: Exploration of collective tactical variables in elite netball: An analysis of team and sub-group positioning behaviours
Source: PLoS One. 2024 Feb 26;19(2):e0295787. doi: 10.1371/journal.pone.0295787 (PMC10896551; doi:10.1371/journal.pone.0295787)
Supplement: Attachment — Submitted filename: Response To Reviewers.docx [file pone.0295787.s039.docx]

**RESPONSE TO REVIEWERS**

| **REVIEWER 1** | | |
| --- | --- | --- |
| **Reviewer Statement** | **Response** | **Line #** |
| L3 – please also state positional spatial restrictions alongside unable to move with the ball. If not space for this, I would remove the bracket as it suggests that is the only restriction. | Thanks for comment, have changed the text to the following:  “(players unable to move when in possession of the ball and positional spatial restrictions)” | 4 |
| L6 – please change national-level to elite. You use elite in the title. Or use professional throughout | Changed manuscript wide to use “elite” and not national-level | 6 |
| L31 – please add a full stop at the end of the sentence. | Fullstop added | 33 |
| L44 – please remove the full stop before the citation. | Thanks, have removed this | 46 |
| L46-47 – should this be the same paragraph? Please address paragraph structure as lines 47-49 shouldn’t be a stand a-lone paragraph. | Thank you for picking this up. Have updated so these are one paragraph. | 49-51 |
| L57 – ‘and in Australian rules..’ should this be ‘in Australian rules..’ | Changed to “in Australian rules” as per your comment. | 58 |
| L63 – please clarify here what you mean by ‘marker of performance’. What performance outcome? | We have changed the use of “marker” to “insight into tactics, positioning and strategies” to be more specific to what collective tactical variables are useful for at the team level. | 64 |
| L68 – please add a full stop at the end of the sentence. | Fullstop added | 69 |
| L68-69 – again, should this be a separate paragraph? | Updated so these are one paragraph. | 72 |
| L74 – please can you expand on why important | We, have added a sentence explaining the importance of mean, variability and irregularity. See below:  “As they provide further context to the range, central point and predictability of collective tactical variables.” | 81-82 |
| L79 – you start to use ApEn here. I assume this is an abbreviation for approximate entropy but you haven’t identified it and then later go back to using the full write out. Please can you clarify in text. | We have changed the text here to ‘approximate entropy’ as that is what is referenced previously. | 88-89 |
| L81 – please add a full stop at the end of the sentence. | Fullstop added | 90 |
| L89 – please add ‘positional’ to the court restrictions statement i.e., positional court restrictions | ‘positional’ added | 98 |
| Lines 96 – 101 – Please make clear in this section that the studies cited aren't in netball - given the paragraph starts on research in netball it reads as if these are in netball too. | Thank you for the suggestion. We have added this in to make it clear the studies are not netball related | 104 |
| Line 102 – could you add a sentence stating these are yet to be investigated in netball. This would lead into the aims better. | Thanks for the suggestion. Have added this in. See below:  “These variables have yet to be studied in netball and thus provide several novel streams of research.” | 114:115 |
| Line 104 – it becomes more clear later in the paper but at this stage it isn’t clear what you mean by sub-groups please make this more clear eg. By stating the groups in brackets. | Groups added here | 119 |
| L111 please amend sentence. You state participants twice. Please also add in that they are females. | Sentence fixed and ‘females’ added | 124 |
| L110-11 as per comment in abstract – clarity and consistency in terminology of language use for playing level. At the moment you have used three different descriptors – national, elite and professional. | Thanks for the suggestion, have updated this to only be elite netball throughout the manuscript |  |
| L120 please can you provide information of positional sub-groups here and sample size for each group. | please refer to the section; data processing where the sub-groups are introduced and have been detailed | 192:196 |
| Event data section  Please refer to and use the recent consensus for definitions - particularly for clarity on use of ‘Turnover’  <https://bjsm.bmj.com/content/early/2023/02/07/bjsports-2022-106187.abstract?casa_token=Tg_fKl9PXFQAAAAA:YIFltSYgiE2-X2AHTLAUc0iyF9UkoKQjaw3L-hYm1uHeX6ygkn17IQTdAbmbJ00aqkZUDIov7iU> | Thank you for this resource, have updated turnover to the include general play turnover and unforced turnover as stated in the resource | 158 |
| L149 – can you give a couple examples of ‘other’ | Have added examples to this section as per your comment | 165-166 |
| Can you provide the R packages used for different stages of analysis. | Have added in the list of R packages used in this section | 178 |
| L170 – please remove the full stop before the citation. | Fullstop removed | 188 |
| L171 – more detail could be provided here on what the points mean – however I am aware it is already a long paper! | Thanks for the suggestion, as you mentioned, it is a lengthy paper already and we would be concerned more information here will add considerably to this length. However if you think it is important the editor might have a suggestion as to how we might address this issue |  |
| L174-176 – positional grouping is unclear. Why have you included WA and WD in multiple groups. You have cited a previous study but they haven’t done this they have actually analysed as separate positions but discussed the, in defensive and attacking groups with Centre as stand alone. Please provide further justification for this. Please also consider the term ‘forwards’ this isn’t really used in current netball literature, could you change to attackers given you have defenders? | Thanks for the comments.  The WA and WD positional restrictions sees them cover both midcourt and either attacking or defensive thirds. Therefore to keep the subgroups even (instead of having 3 mid, 2 fwd and 2 def) we decided to have the WD and WA span two subgroups. From a collective tactical variable perspective this is also important as these player take affordance from both of their related subgroups whereas the other players are mostly active in only one sub-group.   The reason we chose forwards was to distinguish between attacking possession and attackers. And decided not to use the term shooters as this only applies to the GS and GA and not the WA |  |
| L238 – ‘were’ used in the analysis | change made | 256 |
| L304 – please ensure consistency in capital or lower case P | Thanks for picking this up - changed throughout the document | 325 |
| You have done a great job at a difficult task of summarising and presenting such a large amount of data. However, I do wonder whether some of what is in supplementary is included in text – particularly the sub-group analysis given it is discussed a lot. Is there any way you could combine some tables/ figures? I appreciate this is easier said than done! | Thanks for the great comment. A lot of time was spent visualizing these results and we trialed many options. The current format was the best way we found to present the most important data with all the contextual information it requires. |  |
| Table 3 and 4. Can you enter a new line post the table title for the information with it to make the table title more clear. | Thanks for the suggestion, have added a line space to make clear the title and subsequent information. | Table 3 and 4 |
| Is the statement on ‘with the exception of the mean centroid….’ Needed given you justify statistics in the methods. | Thanks, we want to keep this here to provide as much context as possible. |  |
| L363 as stated above given the aim was to investigate sub-groups and team I would like to see more results for the sub-groups in-text not supplementary. | Thanks for the comment. With the expansive number of results we have due to the team and also three sub-groups it was an important decision, as to which results we discussed and displayed in the main text vs the supplementary. For this reason we selected the most interesting and key results and for further results readers are welcome to explore the supplementary material. |  |
| L393 if tables have to remain supplementary could you provide some data in text to support. | Due to large amount of numbers and statistical results presented in the tables, in the results section we utilized the findings decision and magnitude to present a clear and clean way of exhibiting the key findings, alongside reference to the tables. I understand we have many results, however by adding further data into the text, we will be doubling up the results in the tables. |  |
| Figures 5-7  You don’t refer to figures 5, 6 or 7 in text in the possession section? This would likely overcome my comment about data in text if there are figures to support as well.  Please also consider merging these into one figure and having as figure 5a,b,c | Have updated the text so we have reference to the figures. Thanks for the suggestion of merging, I have previously tested this however to ensure the integrity and quality of the figures, we were not able to fit them on one landscape page without compromising the quality. | 414 |
| L463 Please consider the use of the word apparently here - if it is evident in the data state that, if it is speculative state that | ß changed it to a speculative response | 487 |
| L468 More explanation here - figure 1 doesn’t show this clearly. Readers who don’t know netball but are reading from a methodological perspective would need to understand the restrictions of these positions.  Also consider here that this is one team only over 7 matches and that they may have been playing a holding GS for example rather than rotating circle (as highlighted earlier as a consideration) - therefore acknowledging a limitation of playing style is important | Thanks for this suggestion. We have added this into the text to communicate the important point you make | 495-496 |
| L472 Please summarise the findings with practical or future research application. It currently isn’t clear to what this means and how it can be used | We have added in the practical reasoning for using these variables and have attempted to make this section more clear | 506-508 |
| L473 Be consistent with terminology below you state positional tactical variables - please do the same here so it is clear discussing the same thing given the large amount of data/variables in the study | Thanks, have change all terminology to be collective tactical variable and consistent throughout manuscript | 509 + 521 |
| L488 Again, this use of apparently isn’t clear | Changed as per your previous comment | 517 |
| L491 So what can coaches do with this? You state most practical for coaches and players in the above paragraph so I would like to see more practical applications provided. | We have added in practical application for coaches. See below:  “For coaches this is may be useful as a benchmark of how certain strategies could be performed.” | 520-521 |
| L524-526 Expand further - therefore less movement off the ball and shorter passes? | Thank you for another excellent addition. Have added this in. | 558-559 |
| L520 – brackets for citation date | Brackets added | 562 |
| L532 please add consideration of different playing styles vs different oppositions based on shooting circle | Thanks, have added this in | 563-564 |
| L533 – you state similar findings have been found but don’t provide a citation – please add. | reference added | 568 |
| L582 – again, how? This would be okay if provided examples in discussion above. | Thanks for this comment have updated | 618 |
