# Supplementary material for: Exploration of collective tactical variables in elite netball: An analysis of team and sub-group positioning behaviours
Source: PLoS One. 2024 Feb 26;19(2):e0295787. doi: 10.1371/journal.pone.0295787 (PMC10896551; doi:10.1371/journal.pone.0295787)
Supplement: Attachment — Submitted filename: Response To Reviewers.docx [file pone.0295787.s040.docx]

**RESPONSE TO REVIEWERS**

| **REVIEWER 1** | | |
| --- | --- | --- |
| **Reviewer Statement** | **Response** | **Line #** |
| Line 76 please address sentence structure – the sentence added doesn’t flow on its own and should be part of the prior sentence. | Thanks good comment. I have reworded the start of the sentence to make it flow correctly. See below:  “*The afore mentioned collective tactical behaviours can provide a global insight (at the team-level) into tactics, positioning and strategies, it has however been suggested that collective behaviours should also be analysed at sub-group levels.”* | 76 - 78 |
| Line 94 consider re wording ‘inability of the player in possession of the ball to move’ – technically they can move, just not replace their landing foot – therefore at the elite level they frequently still move 1m or so in possession of the ball | Thank, great point. Have updated this to explain the restriction on movement, and specifically regarding the landing foot replacement:  “*This is pertinent to netball, as the positional court restrictions and restriction of the player in possession of the ball to move (not allowed to replace landing foot once in possession),”* | 109-110 |
| L169 space needed between . and utilizing. | Thanks, this has been updated. | 187 |
| L186 I understand your response to my comment about the positional sub-groups, however this statement is still incorrect as it the groups you have used and how you have termed them isn’t in-line with previous research with some positions in multiple groups and attackers being termed forwards. Please either adapt this sentence to acknowledge this. | Thanks for the comment. I understand the perspective regarding sub-groups. I have updated the paragraph top acknowledge it is not exactly like previous work and have also outlined the justification for using different terminology:  *“These positional sub-groups were selected to mirror previous research (69) as close as possible; previously forwards were termed ‘attackers’, however in this research they were termed forwards to avoid confusion between attacking possession. Additionally sub-groups contain* *positional overlap (wing attack part of forwards and midcourts and wing defence part of defenders and midcourts), this was to ensure at least three players in each group for calculation purposes.*” | 204-210 |
| L472 ‘subgroups’ – throughout the rest of the paper it is written as sub-groups except this sentence. Please change and check the rest of the manuscript. | Good pickup. Have updated all mention to be “sub-groups” | Whole paper |
| L543 – please check the flow of the last two sentences and consider combining them. | Thanks for this suggestion. I agree and have merged the two final sentances into one so they flow better:  “*Finally, exploration at the team and sub-group level, has displayed interactions among each of their components and how specific positions can influence organisation levels of higher order, providing an initial base of knowledge to support the implementation of this type of analysis in elite netball.”* | 617-620 |
